# Single-Step Synthesis of Cs_3_Bi_2_I_9_ Nanocrystals for Scalable Direct X‑ray Detectors

**DOI:** 10.1021/acsenergylett.5c02509

**Published:** 2025-11-11

**Authors:** Ramavath Babu, Joydip Ghosh, Nadine J. Schrenker, Kavya Reddy Dudipala, Yi-Teng Huang, Yixin Wang, Shiling Dong, Deepika Gaur, Sara Bals, Sergio Gómez-Graña, Xian Wei Chua, Isabel H. B. Braddock, Matthew. C. Veale, Matthew. D. Wilson, Jack Matthew Woolley, Akshay Rao, Robert L. Z. Hoye, Lakshminarayana Polavarapu

**Affiliations:** † CINBIO, Universidade de Vigo, Materials Chemistry and Physics Group, Department of Physical Chemistry, Campus Universitario Lagoas Marcosende, 36310 Vigo, Spain; ‡ Inorganic Chemistry Laboratory, University of Oxford, Oxford OX1 3QR, United Kingdom; ■ Electron Microscopy for Materials Science (EMAT) and NanoLight Center of Excellence, University of Antwerp, Groenenborgerlaan 171, 2020 Antwerp, Belgium; § UKRI Science & Technology Facilities Council, 97008 Rutherford Appleton Laboratory, Didcot, Oxfordshire OX11 0QX, United Kingdom; ⊥ Department of Chemistry, 2707 University of Warwick, Gibbet Hill Road, Coventry CV4 7AL, United Kingdom; £ Department of Chemical Engineering and Biotechnology, 2152 University of Cambridge, Philippa Fawcett Drive, Cambridge CB3 0AS, United Kingdom; $ Cavendish Laboratory, 2152 University of Cambridge, JJ Thomson Ave, Cambridge CB3 0HE, United Kingdom

## Abstract

Lead-free perovskite-inspired materials have emerged as promising candidates for direct X-ray detection. However, in the early exploration of emerging materials, the focus was on large single crystals. Herein, we report a facile, scalable, single-step synthesis of high-quality Cs_3_Bi_2_I_9_ nanocrystals (NCs) directly from their precursor powders through an ultrasonication approach. The large-scale synthesis of the NCs allowed for the production of 0.78 cm^2^ pellets used in the fabrication of X-ray detection devices, which exhibit a high bulk resistivity of 1 × 10^11^ Ω cm and a low dark current density of 3.3 nA cm^–2^ under an applied bias of 50 V (357 V cm^–1^ electric field). These devices achieve a limit detection of 108 nGy_air_ s^–1^, an order of magnitude improvement over the a-Se used in commercial medical imaging, along with stable current under continuous X-ray exposure with a peak energy of 35 keV_p_. Finally, we demonstrate the scale-up of these detectors by producing thick films 9 cm^2^ in area, achieving a performance comparable to that of the detectors based on pellets.

## Graphical abstract

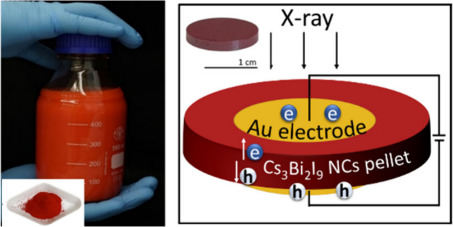

Lead halide perovskites have received increasing attention and have already been found promising for a wide range of optoelectronic applications, including photovoltaics,^1,2^ light-emitting diodes (LEDs),^3^ lasers,^4^ and photodetectors,^5^ owing to their high charge-carrier mobilities,^6,7^ and long diffusion lengths.^6,8^ In addition, one of the most promising applications of these materials, now approaching practical implementation, is in radiation detection, thanks to their exceptional ability to attenuate high-energy electromagnetic radiation.^9–15^ However, due to concerns about toxicity and long-term stability, Bi-based perovskite-inspired materials have emerged as promising alternatives.^16,17^ The replacement of Pb^2+^ with Bi^3+^ leads to low-dimensional metal halides (A_3_Bi_2_X_9_, where A = CH_3_NH_3_^+^ (MA^+^) and Cs^+^; X = halides) or double perovskites with an additional monovalent ion incorporated (A_2_BiAgX_6_).^18,19^ In zero-dimensional (0D) structures with the general formula A_3_Bi_2_X_9_, BiX_6_ units arrange into face-sharing octahedral pairs that are isolated from each other.^20^ Despite their low electronic dimensionality, perovskite-inspired single crystals (SCs) can exhibit high charge-carrier mobilities and long lifetimes. For example, Cs_3_Bi_2_I_9_ single crystals have been reported to achieve electron and hole mobilities of 4.3 and 1.7 cm^2^ V^–1^ s^–1^, respectively, along with prolonged charge-carrier lifetimes of approximately 11 μs.^21^ The presence of heavy elements Bi and I gives Cs_3_Bi_2_I_9_ a high average atomic number (62.3), and coupled with a high mass density (5.02 g cm^–3^), this enables strong attenuation of ionizing radiation.^16,22–24^ Consequently, extensive research has been made to optimize the synthesis of centimeter-sized single crystals.^22,23,25–28^ However, these processes present significant challenges, including the need for high processing temperatures (100–350 °C),^26,29,30^ large quantities of precursors (in grams),^29,30^ and prolonged processing times (2–30 days).^29–32^ Alternatively, colloidal NCs can offer solution-processability and could offer a more scalable route to fabricating thick films or pellets.

Colloidal lead halide perovskite NCs and their derivatives have shown great promise for scintillation-based X-ray detection, where detection occurs through their fast and bright radioluminescence in response to X-ray excitation.^9–12,33–35^ Although Bi-based perovskites and their derivatives are typically weakly luminescent or nonemissive, limiting their effectiveness for scintillation-based X-ray detection, they hold promise for direct X-ray detection by converting incoming X-ray photons directly into charge carriers. However, this capability has not yet been demonstrated in the nanocrystalline form. For applications in X-ray detectors, large-scale synthesis routes are essential not only for cost-effectiveness but also for fabricating large-area X-ray detectors that are sufficiently thick for effectively attenuating high-energy X-rays.^36^ The 0D Cs_3_Bi_2_I_9_ NCs have been synthesized by employing techniques like hot-injection and ligand-assisted reprecipitation (LARP).^37–41^ Although hot-injection synthesis yields uniform NCs, they often require inert conditions and presynthesis of precursors, which pose significant challenges for large-scale production. Developing large-scale synthesis routes that are readily compatible with the formation of samples >200 μm in thickness is critical for high-energy X-ray detector applications. For instance, a 196 μm thick film of Cs_3_Bi_2_I_9_ is required to attenuate 90% of incident X-rays at an energy of 35 keV.^24,42^ To date, the X-ray detection performance of Cs_3_Bi_2_I_9_ NCs has not been demonstrated. Compared with previous reports, our approach offers two key advances: (i) scalable synthesis compatible with the fabrication of thick films and pellets and (ii) demonstration of a large-area detector (active area of 0.25 cm^2^ and film area of 9 cm^2^), which exceeds typical device dimensions reported in the literature, while maintaining stable performance.^43,44^

In this work, we report a facile, scalable, single-step synthesis of high-quality colloidal Cs_3_Bi_2_I_9_ NCs directly from their precursor powders by ultrasonication under ambient conditions. This approach enables gram-scale production of uniform nanocrystals with a high reaction yield of 86% by a simple, rapid, and sustainable manner, without the need for an inert atmosphere or complex equipment. They exhibit a hexagonal crystal structure with a *P*6_3_/*mmc* space group, as confirmed by high-resolution high-angle annular dark-field scanning transmission electron microscopy (HAADF-STEM) imaging and X-ray diffraction (XRD) patterns. The charge-carrier dynamics of the NCs were studied by transient absorption spectroscopy. Benefiting from large-scale synthesis of Cs_3_Bi_2_I_9_ NCs that resulted in high yields of NC powders after solvent evaporation, we fabricated a scalable X-ray detector from a Cs_3_Bi_2_I_9_ pellet pressed from NCs powders and demonstrated its X-ray detection performance using an X-ray source with peak energy of 35 keV_p_ with stable current during 25 min of continuous X-ray exposure.

The Cs_3_Bi_2_I_9_ NCs were synthesized from the corresponding precursors using a direct tip-sonication method that was previously reported for Pb and Sn-based colloidal perovskite NCs, as illustrated in Figure 1a.^45,46^ In the typical synthesis, the precursor salts (Cs_2_CO_3_ and BiI_3_) and capping ligands (oleic acid and oleylamine; OA and OLm, respectively) were added to octadecene (ODE) in a 250 mL reagent bottle and then subjected to tip-sonication. Initially, the solution appears black; however, with continued sonication, it gradually transitions to an orange color (Figure 1b). The method relies on sonication to induce the formation of a cesium oleate complex that reacts with BiI_3_ in the presence of OA and OLm to produce colloidal Cs_3_Bi_2_I_9_ NCs all in one step and in one pot. These NCs were subsequently purified by centrifugation and dispersed in hexane, as shown in Figure 1b. Transmission electron microscopy (TEM) reveals that the NCs exhibit hexagonal and truncated triangular (irregular hexagonal) morphologies with near-monodisperse size distribution (Figure 1c and Figure S1, Supporting Information). The average size of the NCs is ∼26 ± 4 nm (see particle distribution in Figure S1, indicating slightly larger than the size previously obtained using the hot injection approach (18–20 nm).^40,47^ The XRD pattern of the as-prepared Cs_3_Bi_2_I_9_ film, obtained by drop-casting NC dispersions, is presented in Figure 1d. The diffraction peaks observed at 12.5°, 21°, 26°, 27.5°, 30°, and 32.5° closely correspond to the characteristic crystalline peaks of Cs_3_Bi_2_I_9_, aligning well with the simulated XRD pattern. Furthermore, the Pawley refinement of the XRD data resulted in an excellent fit with a low residual (Rwp = 3.7%), confirming that the NCs are highly crystalline and phase-pure (see Figure S2, Supporting Information).

**Figure 1:**
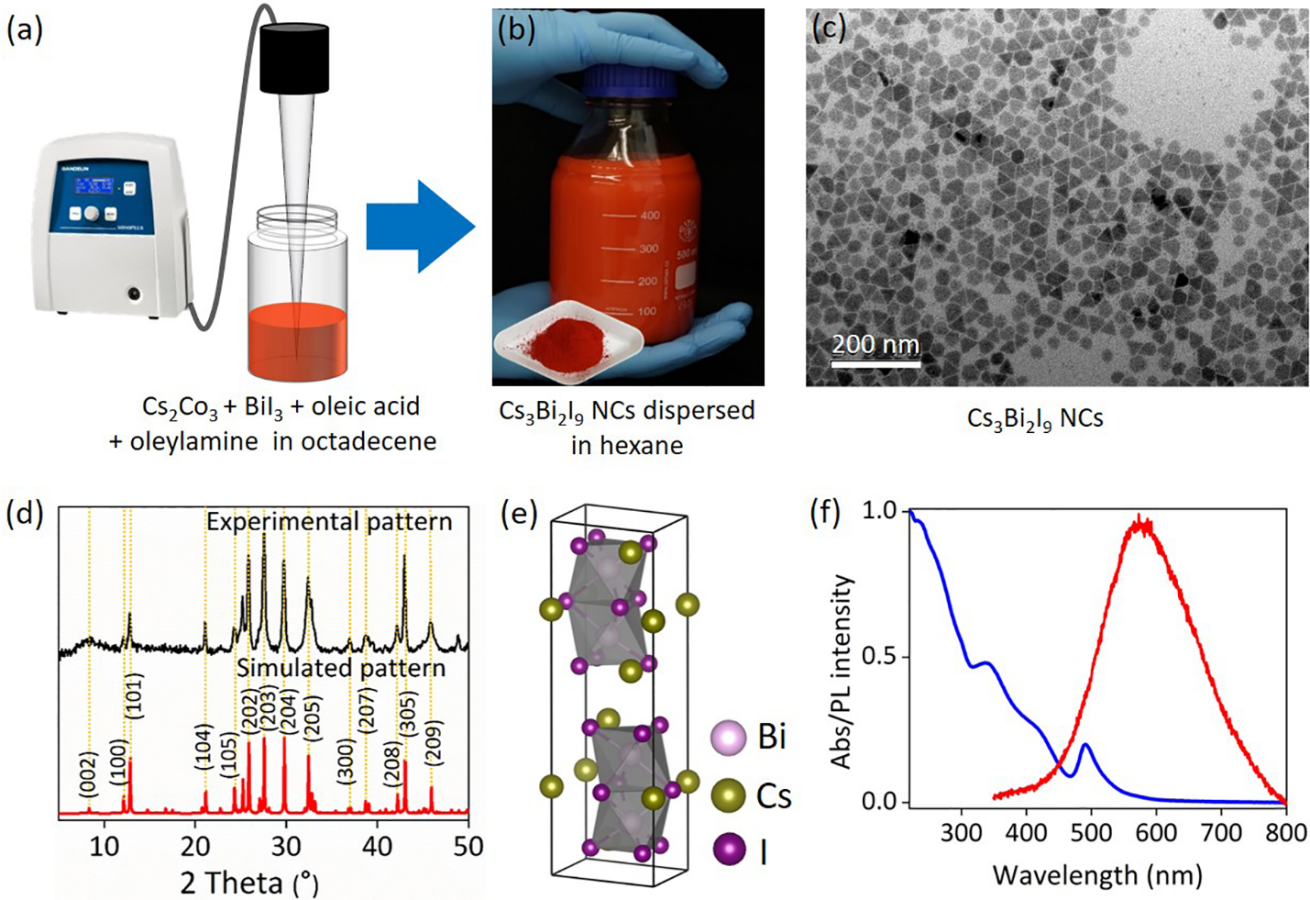
Synthesis, crystal structure, and characterization of 0D Cs_3_Bi_2_I_9_ NCs. (a) Single-step synthesis approach through tip-sonication. (b) Image of large-scale Cs_3_Bi_2_I_9_ NCs dispersed in hexane and crystalline powder of the NCs (insert). (c) TEM image of Cs_3_Bi_2_I_9_ NCs, showing a heterogeneous mixture of hexagonal and truncated triangular NCs with an average diameter range 25–30 nm. (d) XRD patterns of the NCs compared with their simulated patterns demonstrated a hexagonal phase with a *P*6_3_/*mmc* space group. The *hkl* values were obtained using the Mercury crystal analyzer software based on the simulated pattern.^26^ (e) Crystal structure of Cs_3_Bi_2_I_9_ perovskite and (f) UV–vis absorption and photoluminescence spectra of the NCs measured in hexane dispersion, and thin-film form, respectively. The UV–visible spectra highlight a negligible scattering tail in the low energy region.

The powder XRD analysis confirms the formation of a well-defined crystalline phase with a typical hexagonal *P*6_3_ */mmc* space group. Structurally, Cs_3_Bi_2_I_9_ consists of BiI_6_ octahedra that share faces to form discrete dimeric [Bi_2_I_9_]^3–^ anionic units (see Figure 1e). This distinctive arrangement results in a 0D molecular crystal structure, where the individual [Bi_2_I_9_]^3–^ units are spatially isolated, leading to unique optoelectronic properties with high exciton binding energy. To further verify the composition, X-ray photoelectron spectroscopy (XPS) analysis was performed. The survey spectrum confirms the presence of Cs, Bi, and I as the only core elements, along with minor C and O signals likely attributed to surface contamination, common for samples processed in air with organic ligands. The high-resolution spectra of Cs 3d, Bi 4f, and I 3d display the expected binding energies, consistent with Cs^+^, Bi^3+^, and I^–^ oxidation states (Figure S3, Supporting Information). Quantitative analysis yielded a Cs:Bi:I ratio of 3.08:2:8.82, which is in close agreement with the ideal stoichiometry of Cs_3_Bi_2_I_9_, confirming the phase purity. Raman spectroscopy further confirms the phase purity of the as grown Cs_3_Bi_2_I_9_ NCs (see Figure S4, Supporting Information), which showed characteristic peaks at 89, 104, 120, and 147 cm^–1^. The modes at 89 (E_2g_, asymmetric) and 104 cm^–1^ (A_1g_, symmetric) are attributed to bridged Bi–I stretching vibrations, while the modes at 120 (E_1g_, asymmetric) and 147 cm^–1^ (A_1g_, symmetric) correspond to Bi–I stretching in the [Bi_2_I_9_]^3–^ anion.^48^ These Raman features confirm the high phase purity of the NCs. Thermal stability was evaluated using thermogravimetric analysis (TGA), which shows that the materials remain stable up to ∼290 °C before major decomposition begins, with further details provided in the Supporting Information (Figure S5).

The UV–visible absorption spectrum of Cs_3_Bi_2_I_9_ NCs, recorded from a hexane dispersion (see Figure 1f), exhibits a distinct peak centered at 490 nm, along with a broad absorption extending down to 300 nm. These broadband features have at least two sub-bands at 420 and 338 nm, which have been attributed to electronic transitions from the ground ^1^S_0_ state to the ^3^P_1_ excited states of Bi^3+^ ion within the isolated [Bi_2_I_9_]^3–^ clusters.^47,49^ The NCs exhibit an indirect band gap of ∼2.15 eV, as determined from the Tauc plot analysis of (*αhν*)^1/2^ versus photon energy (*hν*) (see Figure S6, Supporting Information). This is in good agreement with previously reported values in the literature.^47,50,51^ Furthermore, the absorption peak at 490 nm is associated with a localized excitonic transition of the [Bi_2_I_9_]^3–^ clusters. The NCs exhibit weak photoluminescence (PL) with a peak centered at 640 nm, indicating a significant Stokes shift relative to the absorption onset, which agrees well with previous reports on polycrystalline thin films.^30,38,47,49,52^ This significant Stokes-shifted emission suggests that it likely occurs through the recombination of trapped carriers, either on the surface or at defect states within the NCs (Figure S7, Supporting Information).^38,47,53^ Time-resolved PL measurements of Cs_3_Bi_2_I_9_ NCs revealed biexponential decay traces with an average lifetime of 0.30 ns (see Figure S8, Supporting Information), which is significantly shorter than that of NCs synthesized via the hot-injection method, as previously reported.^39^ This rapid decay component is likely attributed to trap-state relaxation.^54^

Figure 2a presents a representative HAADF-STEM image of the as-synthesized Cs_3_Bi_2_I_9_ NCs, highlighting their characteristic truncated triangular morphology alongside nanocrystals with hexagonal shapes. Atomic-resolution HAADF-STEM images of a Cs_3_Bi_2_I_9_ NC, acquired along the [001] zone axis (ZA), confirms its single-crystalline nature and hexagonal crystal structure as shown in Figure 2b,c. Additionally, the fast Fourier transform (FFT) of high-resolution STEM images agrees with the hexagonal Cs_3_Bi_2_I_9_ phase along the [001] zone axis (see Figure S9, Supporting Information). This observation is in good agreement with the single crystal structure Cs_3_Bi_2_I_9_ with the *P*6_3_/*mmc* space group,^20,40,51,55^ which features two pairs of face-sharing halide octahedra in each unit cell with one Bi^3+^ ion in each octahedron, as shown in Figure 2f. The bioctahedra are separated by Cs^+^ cations and align to create a layered structure. When viewed along the hexagonal axis, these bioctahedral units arrange into a honeycomb pattern, as depicted in Figure 2e. Generally, Cs_3_Bi_2_I_9_ NCs are known to crystallize in either hexagonal or monoclinic polymorphs of the perovskite crystal lattice.^47^ The high-resolution HAADF-STEM images revealed that the NCs with truncated triangular and hexagonal morphologies both have a hexagonal crystal structure. Furthermore, Figure S9 in the Supporting Information illustrates the atomic structure along the [100] ZA. Energy dispersive X-ray (EDX) mapping confirms the stoichiometric composition of Cs, Bi, and I according to Cs_3_Bi_2_I_9_ NCs (see Figure S10, Supporting Information).

**Figure 2:**
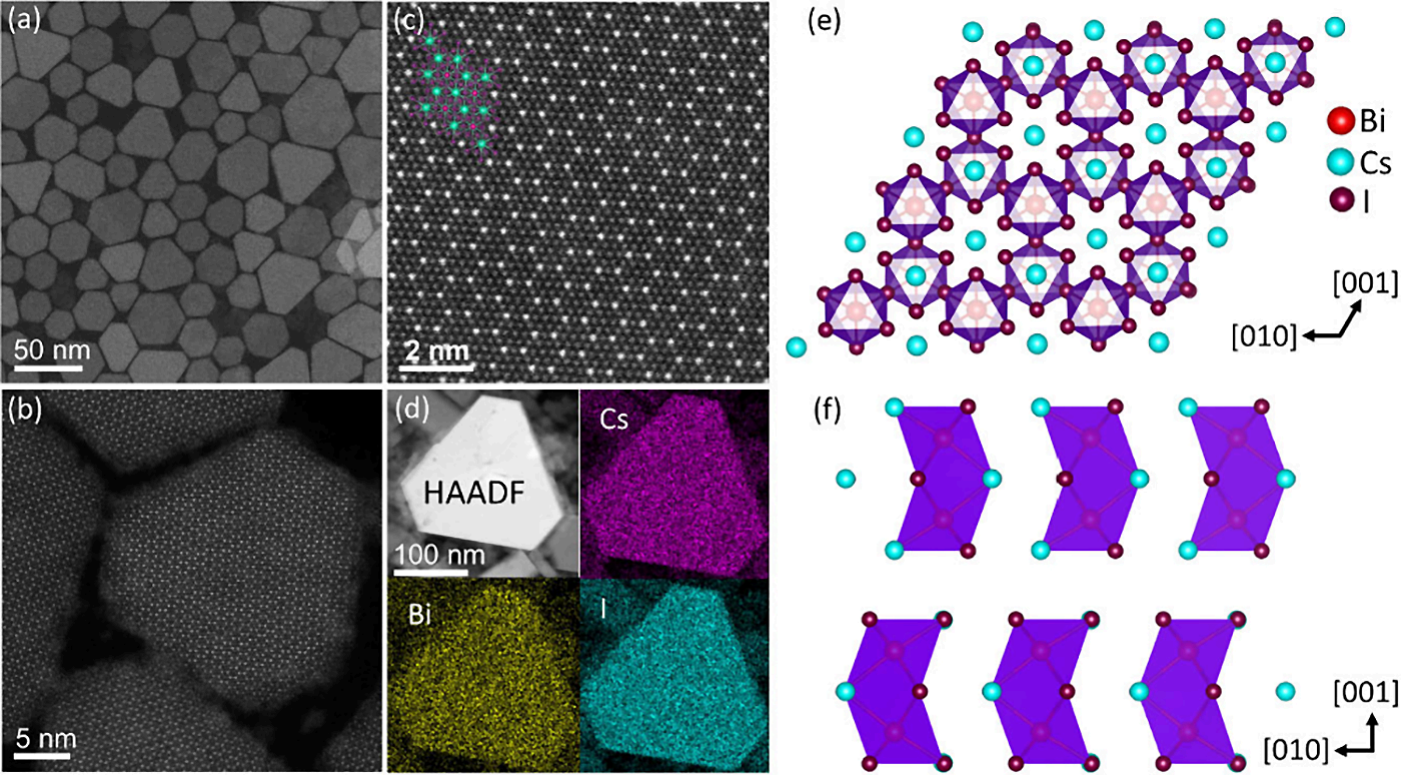
High-Resolution Scanning Transmission Electron Microscopy (STEM) images of Cs_3_Bi_2_I_9_ NCs along with their 3D crystal structure model. (a) Overview STEM images of Cs_3_Bi_2_I_9_ NCs. (b) STEM image of a single Cs_3_Bi_2_I_9_ NC. (c) High-resolution STEM image with an overlay of the crystal structure along the [001] direction. (d) EDX elemental mapping for Cs, Bi, and I (see Figure S10 for the EDX spectrum). (e) 3D representation along the [001] direction, showcasing the hexagonal arrangement of octahedral columns. (f) 3D model of the *P*6_3_/*mmc* crystal structure viewed along the [100] direction, highlighting the spatial separation of bioctahedral units.

Based on the initial analysis, Cs_3_Bi_2_I_9_ NCs showed mixed morphology including both hexagonal and truncated triangular shaped NCs along with a strong scattering tail in the UV–vis spectra (Figure 3a,d). This observation is likely influenced by the precursor ratio (Cs_2_CO_3_/BiI_3_) as a 1:3 molar ratio that is typically employed for Pb-based perovskite NCs. However, the ideal stoichiometric molar ratio of Cs and Bi in Cs_3_Bi_2_I_9_ is 3:2, which requires a molar ratio of 1.5:2 precursors to achieve phase pure NCs. Therefore, the BiI_3_ amount was systematically reduced from 0.3 to 0.254 mmol in a series of incremental steps (2%, 4%, 6%, 8%, 10%, 12%, and 15%) relative to the initial amount to optimize the precursor ratio. As the BiI_3_ amount decreases, the uniformity of hexagonal-shaped NCs gradually improves, while the presence of truncated triangular NCs reduces (see Figure 3b and Figure S11b; size distribution in Figure S12). Interestingly, the scattering tail in the corresponding UV–vis spectra significantly reduced, indicating an improved uniformity of the NCs, as shown in Figure 3e and Figure S11e. It is noteworthy that monodisperse, hexagonal-shaped NCs, with only a minimal presence of truncated triangular variants, were obtained at an optimal BiI_3_ amount of 0.270 mmol (closely matching the theoretical value of 0.266 mmol). These NCs exhibited negligible scattering in the low-energy region of the extinction spectra and sharper, more well-defined diffraction peaks, reflecting improved crystallinity and reduced size-related peak broadening (Figure 3b and Figures S11b and S13). This well-defined morphology and optical quality were preserved even after a 10-fold scale-up, as confirmed by TEM and absorption spectra (Figure S14, Supporting Information). However, below the optimal BiI_3_ amount, hexagonal NCs showed increased size variation, and truncated triangular NCs began to appear (see Figure S11c, Supporting Information). This size variation further increased with decreasing BiI_3_ amount, resulting in polydisperse NCs with broad absorption features (see Figure 3c,f, and Figure S11f; size distribution in Figure S12), although the majority of NCs remained hexagonal as confirmed by statistical analysis (Figure S15, Supporting Information). This increased size dispersion is likely driven by Ostwald ripening,^42,56^ a process in which smaller particles dissolve and redeposit onto larger ones, leading to greater size disparity.

**Figure 3:**
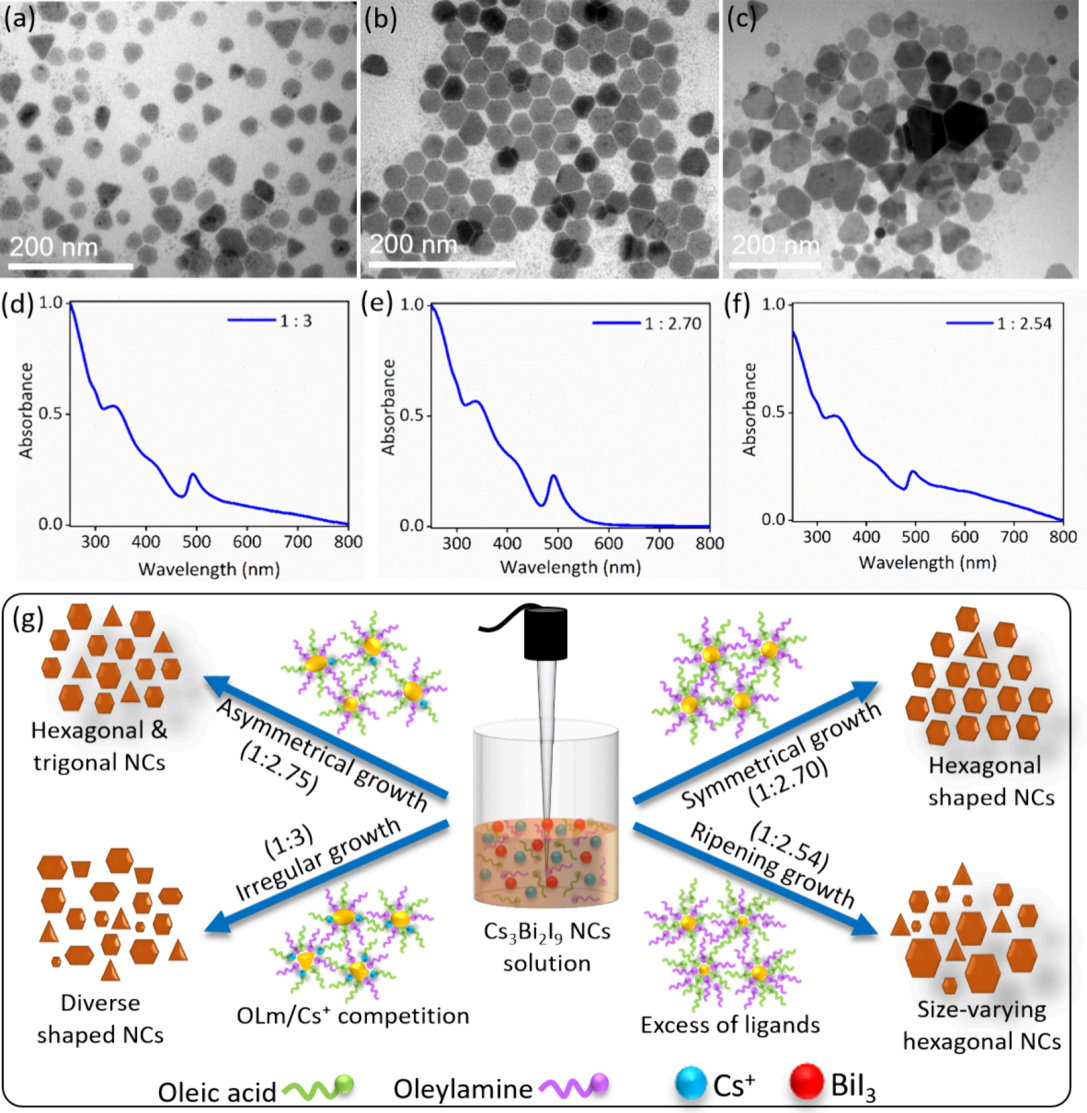
Optimization of Cs_3_Bi_2_I_9_ NCs by varying the BiI_3_ molar ratio, along with their corresponding UV–vis spectra and the proposed mechanism. (a) TEM image of Cs_3_Bi_2_I_9_ NCs synthesized with a Cs_2_CO_3_:BiI_3_ molar ratio of 1:3 and (d) the corresponding UV–vis spectra. (b) TEM image of Cs_3_Bi_2_I_9_ NCs synthesized with a Cs_2_CO_3_:BiI_3_ molar ratio of 1:2.70 and (e) the corresponding UV–vis spectra, indicating negligible light scattering at lower BiI_3_ ratios. (c) TEM image of Cs_3_Bi_2_I_9_ NCs synthesized with a Cs_2_CO_3_:BiI_3_ molar ratio of 1:2.54 and (f) the corresponding UV–vis spectra, suggesting increased light scattering when the BiI_3_ ratio is further reduced. (g) Proposed mechanism for formation of Cs_3_Bi_2_I_9_ NCs with various sizes and shapes at different BiI_3_ concentrations.

Following these observations, we propose a growth mechanism for the NCs, in which the dissolution of BiI_3_ precursors in the presence of OA and OLm ligands plays a crucial role, as depicted in Figure 3g. As noted earlier, the volumes of OA and OLm were kept constant, while the amount of BiI_3_ was varied from 0.30 to 0.254 mmol in incremental steps of approximately 2% relative to the initial amount. The initial precursor ratio of 2:3 creates a Bi rich environment relative to Cs, while 0.5 mL volumes of OA and OLm ligands appear to be optimal, as later confirmed through ligand volume variation studies (see Figures S16–S19, Supporting Information). During sonication, Cs oleate likely forms first and reacts subsequently with BiI_3_, initiating the nucleation and growth of Cs_3_Bi_2_I_9_ NCs. In this process, the OA amount decreases slightly, whereas OLm remains in slight excess, predominantly in its protonated form as 1° ammonium salts. This oleylammonium-rich environment may promote anisotropic growth through competition with Cs^+^ ions, leading to irregular NC morphologies comprising both hexagonal and truncated triangular shapes of varying sizes (Figure 3a). A similar anisotropic growth behavior was reported for CsPbBr_3_ NCs synthesized via hot-injection method under oleylammonium-rich conditions, resulting in nanocubes ranging from 4 to 16 nm in size.^56^ However, decreasing the BiI_3_ amount down to 0.30 mmol, reduces the anisotropic growth, likely due to sufficient ligand availability that promotes isotropic, uniform NC growth (Figure 3b). Below this optimal amount of BiI_3_, excess ligands can trigger Ostwald ripening, where smaller NCs dissolve and redeposit onto larger ones, causing increased size variation among hexagonal NCs (Figure 3c and Figure S11c). This size variation becomes more pronounced with further BiI_3_ reduction, likely due to increased ligand concentration enhancing the Ostwald ripening effect. As previously reported for Cs_3_Bi_2_I_9_ NCs, which undergo not only anisotropic growth but also Ostwald ripening in a ligand-rich environment at 27 °C, transforming nanocubes into larger hexagonal-shaped Cs_4_PbBr_6_ NCs within 10 min.^56^ In addition, Ostwald ripening has also been observed in Pd NCs, where the effect was studied as a function of precursor amount and seed size.^57^ At lower amount and smaller seed sizes, Pd NCs exhibited pronounced Ostwald ripening, while those particles larger than 5.7 nm showed broader size distributions due to simultaneous redeposition onto both smaller and larger NCs. These previous studies of colloidal NC growth dynamics align well with our proposed mechanism, particularly in ligand-rich samples at room temperature, as evidenced by our TEM analysis. Additionally, we investigated the influence of ligand concentration and the temporal evolution of NCs using the optimized Cs_2_CO_3_:BiI_3_ molar ratio of 1:2.70 (Figures S16–S21, Supporting Information). Ligand variation studies confirmed their pivotal role in controlling NC size and promoting uniform, hexagonal-shaped NCs with reduced size dispersion, while time-dependent analysis provided insights into the sequential nucleation and growth processes (Figures S20 and S21, Supporting Information).

With the successful synthesis of uniform and highly crystalline Cs_3_Bi_2_I_9_ NCs, it is essential to investigate their optoelectronic properties, as these directly impact their performance in X-ray detectors. We verified from fitting the absorption spectrum with an Elliott model that the material is highly excitonic, which a high exciton binding energy of 191 meV (Figure 4a).^58^ This is in good agreement with previous reports, and is high, likely because of the confinement of excitations within the quasi 0D bioctahedral units.^38^

**Figure 4:**
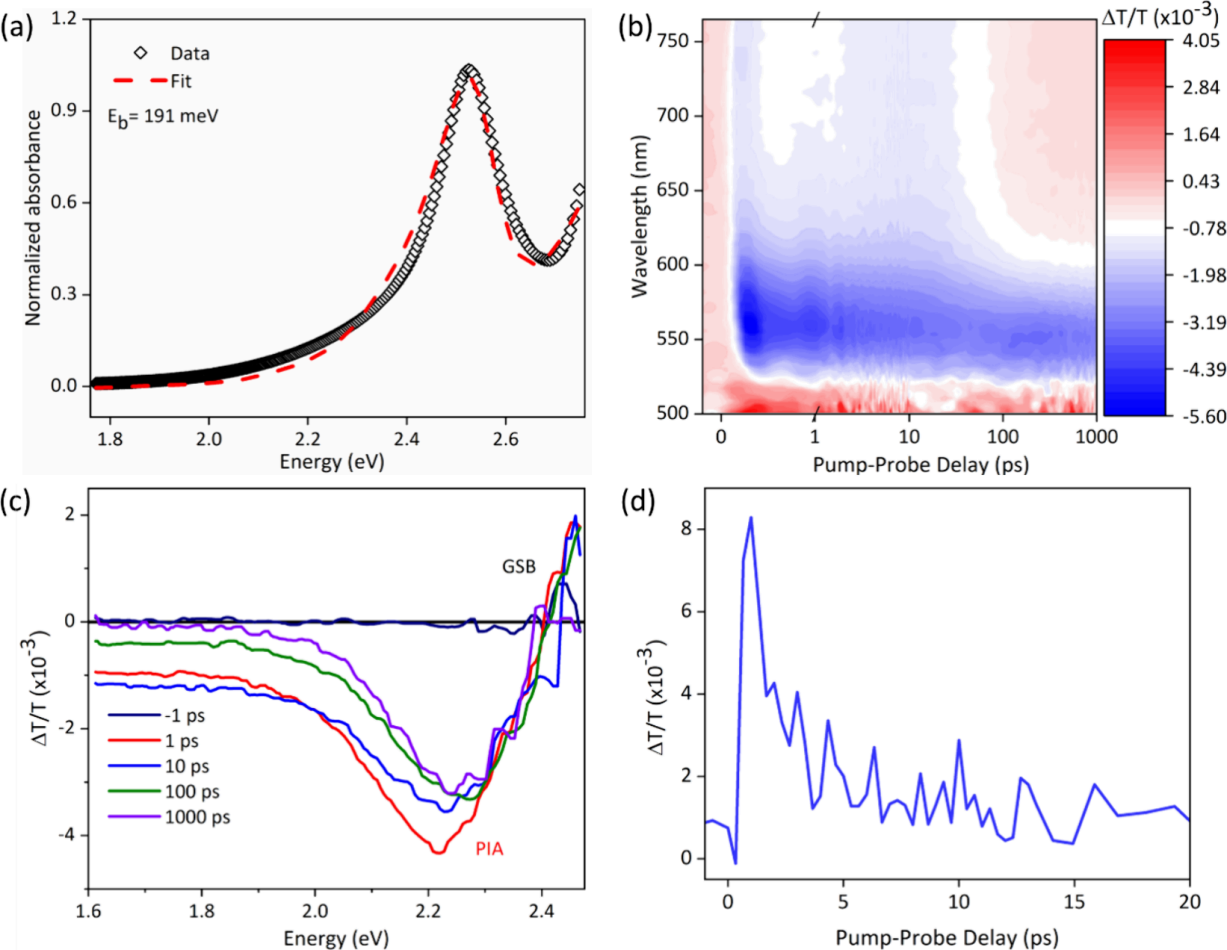
Optical and photoinduced charge carrier dynamics studies of Cs_3_Bi_2_I_9_ NCs. (a) Elliott model fitting of the absorbance of Cs_3_Bi_2_I_9_ NCs to extract exciton binding energy. (b) Ultrafast 2D TA map of Cs_3_Bi_2_I_9_ NCs film under excitation of 400 nm with fluence of 17.3 μJ cm^–2^ pulse^–1^. (c) TA spectrum of the NC films with 400 nm wavelength pump excitations at pump–probe delays of 1, 10, 100 and 1000 ps. (d) OPTP photoconductivity transients of Cs_3_Bi_2_I_9_ NCs film, measured with pump wavelength of 400 nm and fluence of 4.2 mJ cm^–2^ pulse^–1^.

To further investigate the carrier dynamics of Cs_3_Bi_2_I_9_ NCs, both transient absorption (TA) and optical pump-terahertz probe (OPTP) measurements were performed. Figure 4b displays the 2D TA color map of the Cs_3_Bi_2_I_9_ NCs film under 3.1 eV pump excitation, at a fluence of 17.3 μJ cm^–2^ pulse^–1^. As can be seen from Figure 4b,c, a broadband photoinduced absorption (PIA) signal spanning from 1.6 to 2.4 eV along with a ground-state bleach (GSB) peaking near 2.5 eV was present after pump excitation, consistent with prior reports.^43^ Considering the close match with the absorption peak in Figure 4(a), the GSB signal could be unambiguously assigned to the direct transition of excitons. On the other hand, the broadband PIA signal could be a convolution of charge-carrier relaxation at the indirect band edge in addition to bandgap renormalization. The former could be verified from the much longer relaxation lifetime of the PIA kinetics compared to the GSB kinetics (Figure S22, Supporting Information), which originates from the slow carrier transition between the indirect band edges. The latter is clearly reflected in the significant blue shift of the PIA peak within around 100 ps (Figure 4c), which is a very slow process compared to that of conventional semiconductors and lead halide perovskites. Since bandgap renormalization results from the interaction among free carriers, the slow bandgap renormalization process could be caused by the low rate of exciton dissociation into free carriers because of the large exciton binding energy (191 meV), as also observed in quasi-2D perovskites.^44,59^ This low rate of exciton dissociation can be further verified from the OPTP kinetics, which directly reflects the time evolution of the free carrier population. As can be seen from Figure 4d, the OPTP kinetics of Cs_3_Bi_2_I_9_ NCs decayed by more than 75% within 5 ps, suggesting that most free carriers tend to bind with each other and form excitons.^59^ As a result, very few free carriers remained after a 20 ps pump–probe delay. Despite the excitonic feature, which also applies in most A_3_Bi_2_X_9_ materials, the carrier transport within Cs_3_Bi_2_I_9_ NCs under the influence of an electric field seems not be impeded significantly, as will be discussed afterward. This result implies that the formed excitons within Cs_3_Bi_2_I_9_ NCs might be easily dissociated by the applied electric field and hence will not degrade performance of X-ray detection.

In contrast to other optoelectronic devices, X-ray detectors require large-scale material synthesis strategies due to their thicker structure for higher attenuation. For example, a 196 μm thick film of Cs_3_Bi_2_I_9_ is required to attenuate 90% of incident X-rays with an energy of 35 keV.^24,42^ Therefore, our synthesis method is highly suitable for large-scale production of materials for X-ray detection, since we can produce sufficient material to make pellets with thicknesses exceeding this minimum requirement rapidly. For example, to fabricate a pellet with a diameter of 10 mm and a thickness of 1.4 mm, 400 mg of Cs_3_Bi_2_I_9_ NCs is required. Using our method, we can produce gram scale of NCs within a synthesis time of 12 min. Due to the composition of the heavy elements Bi and I, Cs_3_Bi_2_I_9_ has an average atomic number of 62.3.^22,24^ The high average atomic number results in high X-ray attenuation, making the bismuth-based 0D metal halides suitable for sensitive X-ray detection. Utilizing simulation data from the NIST XCOM database, we found that Cs_3_Bi_2_I_9_ demonstrates superior X-ray attenuation compared to other lead halide perovskite materials, Si and CdTe (Figure 5b).^42^ Cs_3_Bi_2_I_9_ NCs powder was pressed into a pellet of thickness 1.4 mm and diameter 10 mm using a hydraulic press. The detailed fabrication steps are illustrated in Figure 5a, and the pellet fabrication procedure is described in the Supporting Information. Furthermore, the X-ray diffraction pattern confirmed that the NCs retained their crystallinity and phase purity during pellet fabrication, with no new diffraction peaks or peak broadening observed after gentle grinding (see Figure S23, Supporting Information). Figure 5c presents a schematic representation of the Au/Cs_3_Bi_2_I_9_ NCs pellet/Au X-ray detector, illustrating the X-ray-induced carrier generation and separation, which contributes to the device’s photocurrent response. The left inset of Figure 5b shows a photograph of the Au/Cs_3_Bi_2_I_9_ NCs pellet/Au detector mounted on a glass substrate with contact pads. The right inset of Figure 5d shows the dark *I*–*V* characteristics of the NCs wafer-based detector. The bulk resistivity of the device was obtained to be 1.02 × 10^11^ Ω cm. The calculated resistivity was significantly higher than that reported for Cs_3_Bi_2_I_9_ polycrystalline wafers and comparable to that of Cs_3_Bi_2_I_9_ SCs (2.79 × 10^10^ Ω cm).^23,60^ The high bulk resistivity of the device results in an exceptionally low dark current density of 2.6 nA cm^–2^ under an applied bias of 28 V (electric field of 200 V cm^–1^), making it well-suited for low-dose X-ray detection and imaging applications.

**Figure 5:**
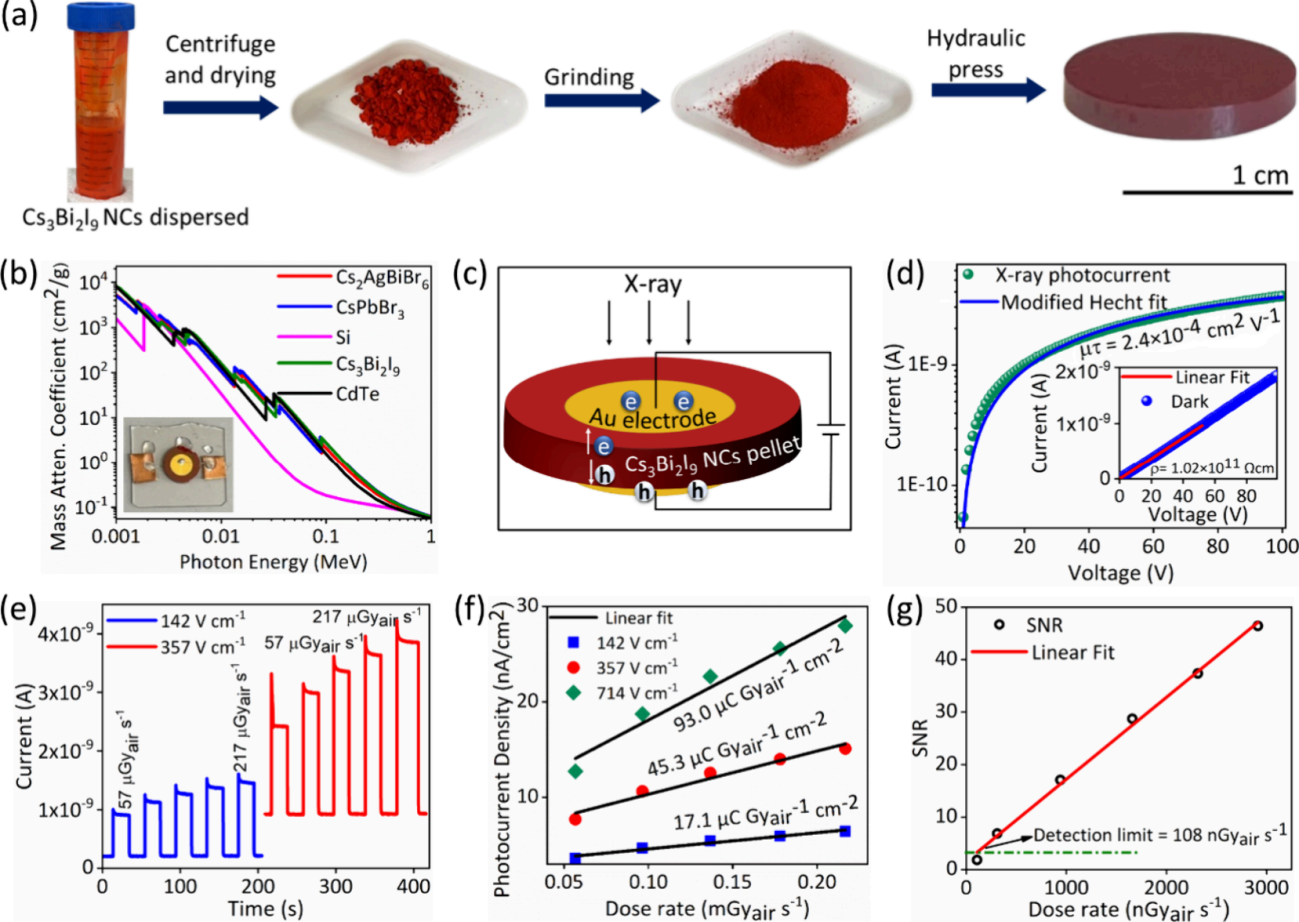
Fabrication and X-ray detector performance of the Cs_3_Bi_2_I_9_ NC pellet-based device. (a) Pellet fabrication involves sequential steps, including drying, grinding, and pressing the powder into a compact form using a hydraulic press. (b) Comparison of mass attenuation coefficients vs photon energy of Cs_3_Bi_2_I_9_ with other perovskites and conventional detector materials. The left inset shows a photograph of the Au/Cs_3_Bi_2_I_9_ NCs pellet/Au X-ray detector. (c) Schematic of the Au/Cs_3_Bi_2_I_9_ NCs pellet/Au X-ray detector. (d) X-ray photocurrent vs applied bias voltage with Hecht fitting. The right inset depicts the dark *I*–*V* of the device and resistivity. (e) Dynamic photocurrent response of the detector under the applied field of 142 V cm^–1^ and 357 V cm^–1^ and varying X-ray irradiation dose rates between 57 and 217 μGy_air_ s^–1^. (f) Photocurrent density vs X-ray dose rates plot of the detector under applied field of 142, 357 and 714 V cm^–1^. The slope of the fit presents the sensitivity of the detector. (g) Comparison of SNR versus irradiation dose rates showing the limit of detection of the detector.

We determined the mobility-lifetime product (μτ) of the NC pellet by using X-ray photocurrent measurements. The μτ product reflects the average distance that photogenerated charge carriers can drift under an electric field before recombining. In our case, the NC pellet contains a significant density of grain boundaries, which may increase nonradiative recombination and scattering compared to SCs. Although α-particle spectroscopy is commonly used to measure μτ values, X-ray-based methods have also been applied, particularly in the context of perovskite SCs, as demonstrated in prior studies.^60,61^ However, it remains an open question as to how well the μτ values obtained via X-ray measurements compare to those derived from α-particle spectroscopy, especially in nanocrystalline systems. For the Cs_3_Bi_2_I_9_ NCs pellet device, the μτ product was extracted using both the modified Hecht fit and a conventional Hecht fit of the photocurrent (dark current subtracted from the total light current) measurement conducted under a range of applied biases, as shown in Figure 5d and Figures S24­(a–b) (Supporting Information). The μτ values are obtained to be 3.6 × 10^–4^ and 2.4 × 10^–4^ cm^2^ V^–1^ using conventional and modified Hecht fit, respectively. The modified Hecht model, which incorporates surface recombination velocity, provides a better fit (Figure S24) which may be due to the nanocrystalline nature of the pellet, where grain boundary recombination plays a significant role.^62^ A detailed description of the fitting procedure is provided in the Supporting Information.

The μτ product was determined to be 2.4 × 10^–4^ cm^2^ V^–1^ (Figure 5d), which is comparable to previously reported values for Cs_3_Bi_2_I_9_ SC and polycrystalline X-ray detectors measured using similar Hecht model analyses.^22,23,60^ We also tested the detector under an Am-241 alpha source to evaluate its suitability for pulse-mode operation. However, no detectable response was observed, likely due to the long carrier drift time (∼300 ms in our pellet, based on reported mobility), which significantly exceeds the typical carrier lifetime.^21^ The dynamic X-ray photocurrent response was evaluated under varying X-ray dose rates, ranging from 57 to 217 μGy_air_ s^–1^, at applied biases of 20 V (electric field of 142 Vcm^–1^) and 50 V (electric field of 357 Vcm^–1^), as shown in Figure 5e. The X-ray photocurrent increased with higher irradiation dose rates due to enhanced photocarrier generation. Additionally, an increase in the applied electric field led to a further rise in photocurrent, attributed to improved carrier separation and transport and a higher charge collection efficiency. Further, we calculated the sensitivity of the Cs_3_Bi_2_I_9_ NCs device from the slope of the photocurrent density versus dose rate, as shown in Figure 5f, obtaining values of 17.1, 45.6, and 93 μC Gy_air_^–1^ cm^–2^ under applied biases of 20, 50 and 100 V, respectively.

To determine the lowest detectable dose rate, the signal-to-noise ratio (SNR) of the NCs pellet detector was plotted against the dose rate, as illustrated in Figure 5g. The limit of detection (LOD) is defined by IUPAC as the dose rate corresponding to an SNR of 3.^63^ For the NC pellet detector, the LOD was found to be 108 nGy_air_ s^–1^, significantly lower than the LOD of a-Se (5.5 μGy_air_ s^–1^) used in flat panel commercial X-ray detectors for medical imaging.^64–66^ Thus, our detector holds promise for lower-energy medical imaging applications, such as mammography and chest X-rays, where improved detection limits can reduce the dose of ionizing radiation to which the patient is exposed, improving the safety of medical imaging. Further, we have tested the operational stability of the device under continuous X-ray on–off states as shown in Figure 6a. The detector demonstrated stable operation, showing no significant change in photocurrent or shifts in dark current during continuous X-ray irradiation for approximately 25 min, confirming the good stability of the device.

**Figure 6:**
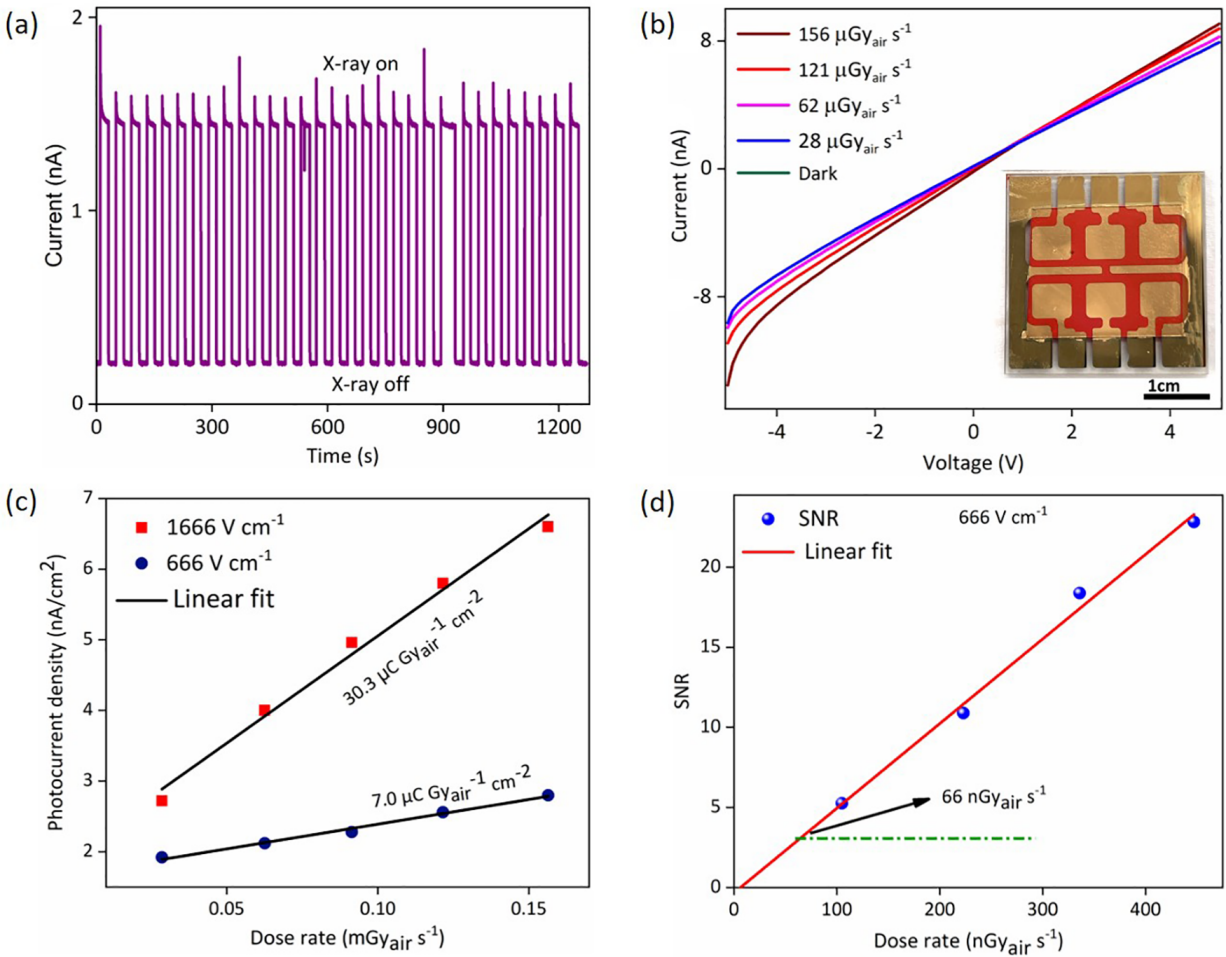
Device performance of Cs_3_Bi_2_I_9_ NCs in large area devices, prepared as pellets and thick films. (a) Operational stability of the pellet X-ray detector under continuous exposure to X-ray on–off states under an applied field of 142 V cm^–1^. (b) *I*–*V* characteristics of FTO/Cs_3_Bi_2_I_9_ NCs thick film/Au detector under dark and X-ray illumination with different dose rates. (c) Photocurrent density vs X-ray dose rates plot of the thick film detector under applied filed of 666 and 1666 V cm^–1^. The slope of the fit presents the sensitivity of the detector. (d) Comparison of SNR vs irradiation dose rates showing the limit of detection of the thick film detector.

Beyond the production of these pellets, we also demonstrated the fabrication of large-area X-ray detectors based on thick films of Cs_3_Bi_2_I_9_ NCs. Such thick films are potentially advantageous for flat-panel imagers in that they can be directly integrated onto the CMOS required, whereas pellets need an adhesive to connect them, increasing the processing complexity and time. A uniform Cs_3_Bi_2_I_9_ NC film with 30 μm thickness was prepared by drop casting the NC ink onto pattern FTO-coated glass substrates 9 cm^2^ in size. A vertical device structure of FTO/Cs_3_Bi_2_I_9_ NCs thick film/Au was fabricated, incorporating six pixels, each with an active area of 0.25 cm^2^, as illustrated in the inset of Figure 6b. Film uniformity was verified through dark *I*–*V* measurements across all six pixels, as shown in Figure S25a, where the consistent *I*–*V* characteristics confirm the uniformity of the thick film. Under one sun illumination, the device exhibited a stable photocurrent response without noticeable current drift under a high electric field of 666 V cm^–1^ (see Figure S25b), highlighting its potential for both visible light and X-ray detection applications. Figure 6b presents the *I*–*V* characteristics of the Cs_3_Bi_2_I_9_ NCs thick-film detector measured in the dark and under X-ray irradiation at varying dose rates ranging from 28 to 156 μGy_air_ s^–1^. As the X-ray dose rate increases, the photocurrent also increases due to enhanced charge-carrier generation from the incident radiation. The temporal photocurrent response at different dose rates under an applied bias of 5 V (corresponding to an electric field of 1666 V cm^–1^) is shown in Figure S26. The detector exhibits X-ray sensitivities of 7 μC Gy_air_^–1^ cm^–2^ at 2 V (electric field of 666 V cm^–1^) and 30.3 μC Gy_air_^–1^ cm^–2^ at 5 V (electric field of 1666 V cm^–1^) bias, respectively (Figure 6c). However, the sensitivity is currently limited by the relatively small active layer thickness of 30 μm. Devices were fabricated using thicker films (∼50 μm), but these thicker films showed cracking and delamination from the substrate. Future efforts to optimize the deposition of NCs to form films exceeding 50 μm (e.g., spray deposition, or screen printing) may help improve X-ray attenuation and sensitivity. To evaluate the LOD of the current devices with 30 μm thick active layers, the SNR was plotted against the X-ray dose rate, as shown in Figure 6d. The LOD was determined to be 66 nGy_air_ s^–1^ under an electric field of 666 V cm^–1^, indicating the detector’s potential suitability for low-dose X-ray imaging applications. This improvement in the LOD for the thick films compared to pellets was due to a reduction in the dark current, likely due to the presence of residual long-chain ligands on the NCs prepared by drop casting. Such ligands, however, are likely detrimental to charge-carrier transport. Further surface engineering of the nanocrystals to strike an improved balance between charge collection efficiency (needed to improve sensitivity) and dark current (to improve LOD) would be needed in the future.

We evaluated both wafer and thick-film devices after three months with a humidity level of ∼20%. Figures S27 and S28 show the X-ray responses of the pellet and thick-film samples after storage. For the pellet, the X-ray sensitivity remained nearly identical to that of the as-grown sample, while the thick-film sample exhibited a slight increase in sensitivity, which may be attributed to strain relaxation or the removal of residual solvent from the film. We have provided a comparison in Table S1 of the Supporting Information. Our pellet- and thick-film-based devices, fabricated using a scalable NC synthesis approach, exhibit comparable or superior performance relative to lead-free, perovskite-inspired polycrystalline materials. A key advantage of our NC ink is that it enables the scalable fabrication of tens of micrometers thick films with continuous morphology.

We demonstrated a facile, scalable, single-step synthesis of high-quality Cs_3_Bi_2_I_9_ NCs directly from precursor powders by the ultrasonication approach. Unlike previously reported approaches that require high temperatures and inert environments, this method enables gram-scale production of uniform NCs under ambient conditions. By systematically varying the precursor ratio and the volume of capping ligands, we achieved precise control over the size and shape of the NCs with reduced scattering. The large-scale synthesis of the NCs enabled the fabrication of pellets and thick films with variable dimensions for direct X-ray detection. The devices exhibited a high bulk resistivity of 1 × 10^11^ Ω cm and a low dark current density of 2.6 nAcm^–2^ under an applied bias of 28 V. Furthermore, it has shown a sensitivity of 93 μC Gy_air_^–1^ cm^–2^, a low detection limit of 108 nGy_air_ s^–1^, and an order of magnitude improved over the commercial flat panel detectors used for medical imaging with high operational stability. These results highlight the potential of using nonfluorescent colloidal halide perovskite NCs for direct X-ray detection, paving the way for future advancements in this area of research.

## Supplementary Material



## Data Availability

Raw data for this paper available from the Oxford Research Archive via the following link: https://doi.org/10.5287/ora-kebqgxzam.

## Notes

Materials and methods, details of the synthesis including optimization and time-controlled synthesis, physical measurements, transient absorption, optical-pump-terahertz-probe measurements, fabrication and characterization of the X-ray detector, Calculation of mobility-lifetime (μτ) using Hecht fitting, size distribution analysis, Raman spectra, XRD, TGA, Tauc plot for indirect band gap estimation, mechanism for radiative recombination, PL decay dynamics spectra, STEM, TEM and SEM images, EDX, thermogravimetry analysis, XPS analysis, TEM of optimization, statistical estimation, digital images, UV–vis spectra, comparison of GSB and PIA, *I*–*V* curves, pump–probe delay curves, photocurrent response analysis graphs, table of detector performance (PDF)

## #

Graduate Institute of Photonics and Optoelectronics, Department of Electrical Engineering, National Taiwan University, Taipei 10617, Taiwan
