# Supplementary material for: Single-Step Synthesis of Cs3Bi2I9 Nanocrystals for Scalable Direct X‑ray Detectors
Source: ACS Energy Lett. 2025 Nov 11;10(12):6092–103. doi: 10.1021/acsenergylett.5c02509 (PMC12706830; doi:10.1021/acsenergylett.5c02509)
Supplement: Supplementary file 1 [file nz5c02509_si_001.pdf]

Supporting Information for:

Single-Step Synthesis of $Cs_3Bi_2I_9$ Nanocrystals for Scalable Direct X-Ray Detectors

Ramavath Babu,[†] Joydip Ghosh,[‡] Nadine J. Schrenker,[#] Kavya Reddy Dudipala,[‡] Yi-Teng Huang,[‡] Yixin Wang,[‡] Shiling Dong,[‡] Deepika Gaur,[†] Sara Bals,[#] Sergio Gómez-Graña,[†] Xian Wei Chua[$,£], Isabel H. B. Braddock,[§] Matthew. C. Veale,[§] Matthew. D. Wilson,[§] Jack Matthew Woolley[⊥], Akshay Rao[$], Robert L. Z. Hoye[‡]* and Lakshminarayana Polavarapu[†]**

[†]CINBIO, Universidade de Vigo, Materials Chemistry and Physics Group, Department of Physical Chemistry, Campus Universitario LagoasMarcosende, 36310 Vigo, Spain.
Email: lakshmi@uvigo.es

[‡]Inorganic Chemistry Laboratory, University of Oxford, Oxford OX1 3QR, United Kingdom.
Email: joydip.ghosh@chem.ox.ac.uk; robert.hoye@chem.ox.ac.uk

[#]Electron Microscopy for Materials Science (EMAT) and NANOlab Center of Excellence, University of Antwerp, Groenenborgerlaan 171, 2020 Antwerp, Belgium

[§]UKRI Science & Technology Facilities Council, Rutherford Appleton Laboratory, Didcot, Oxfordshire, OX11 0QX, United Kingdom

[⊥]Department of Chemistry, University of Warwick, Gibbet Hill Road, Coventry CV4 7AL, United Kingdom

[£]Department of Chemical Engineering and Biotechnology, University of Cambridge, Philippa Fawcett Drive, Cambridge CB3 0AS, United Kingdom

[$]Cavendish Laboratory, University of Cambridge, JJ Thomson Ave, Cambridge CB3 0HE, United Kingdom

Materials:

All chemicals were used as received without any further purifications. Cesium carbonate (Cs_2CO_3, 99%), Bismuth (III) iodide (BiI_3, 99%), Bismuth (III) bromide ($BiBr_3$, 98%), 1-octadecene (technical grade 91%), oleic acid (technical grade 90%), oleylamine (technical grade 70%), hexane (HPLC, grade ≥97.0%, GC). All chemicals were purchased from Sigma-Aldrich.

Synthesis of $Cs_3Bi_2I_9$ NCs: In a typical synthesis, 10 mL of octadecene, along with 0.5 mL of oleic acid and 0.5 mL of oleylamine, was added to precursor powders of Cs_2CO_3 (0.1 mmol) and BiI_3 (0.3 mmol). The reaction mixture was then subjected to tip sonication using a SONOPULS HD 3100 (BANDELIN) at a power of 30 W for 12 minutes. Initially, the solution appeared black; however, as the reaction progressed, its color gradually transformed into orange, indicating the successful formation of $Cs_3Bi_2I_9$ nanocrystals (NCs). The as-prepared NC dispersions were purified by centrifugation at 10000 rpm for 10 minutes to remove unreacted precursors. The resulting NC precipitates were then redispersed in 5 mL of hexane under mild sonication. To further refine the NC dispersions, an additional centrifugation step was performed at 5,000 rpm to remove larger NCs, after which the purified NCs were redispersed in 5 mL of hexane for subsequent studies.

Optimization of $Cs_3Bi_2I_9$ NCs: The synthesis procedure is the same as above, except for the use of varying BiI_3 precursor ratios ranging from 0.30 mmol to 0.254 mmol and different ligand volumes from 0.3 mL to 0.8 mL. A uniform size distribution and excellent monodispersity of hexagonal-shaped NCs were achieved at a BiI_3 precursor ratio of 0.270 mmol with 0.5 mL of capping ligands.

Time-controlled synthesis of $Cs_3Bi_2I_9$ NCs: The synthesis procedure was the same as described above, except that the optimized BiI_3:Cs_2CO_3 molar ratio was used. Aliquots of 1 mL were collected at 2, 3, 4, 6, 8, and 10 minutes, followed by centrifugation and dispersion in hexane.

Physical measurements: The UV-vis absorption spectra of $Cs_3Bi_2I_9$ colloidal suspensions were measured by using Cary 60 (Agilent Technologies). The morphology of obtained particles was characterized using JEOL JEM-1011 TEM operating at an accelerating voltage of 80-100 kV under the vacuum level ~ 3×10^{-5} Pa. Scanning Transmission Electron Microscopy (STEM) images were acquired with a probe-corrected Titan Themis and a cubed Titan Thermo Fisher Scientific Microscope operating at 300 kV with a probe semi-convergence angle of ~ 20 mrad. Energy Dispersive X-ray spectroscopy (EDX) was performed using a Super-X detector and a current of 50pA. Thin-film X-ray diffraction (XRD) patterns were collected on PANalytical Empyrean XRD equipment in the reflection mode using a zero background sample holder. Incident radiation was generated using a CuKα source, λ = 1.5406 Å and data were collected in the 2θ range 5 to 60° in continuous scanning mode with a step size of 0.026° using a Pixel 3D detector. X-ray photoelectron spectra were acquired using a ThermoFisher K-Alpha spectrometer equipped with a hemispherical electron analyzer and a microfocused monochromatic Al Kα X-ray source (1486.6 eV). Thermogravimetric analysis (TGA) was performed using a SETSYS Evolution 1750 instrument (Setaram) under nitrogen atmosphere at a heating rate of 5 °C/min. The Raman measurement was performed under laser excitation of 785 nm. The confocal micro-photoluminescence (µPL) measurements were made using 1ps pulses from a tuneable Ti:sapphire laser. A 36 achromatic microscope objective lens was used to focus the laser and acquire optical images. A frequency-tripled mode-locked 266 nm laser at 76 MHz was directed onto the sample to an excitation spot size approximately 2 µm. The frequency-doubled 400 nm laser was focused to a spot size of 1 µm. Time resolved

photoluminescence (TRPL) measurements were carried out using the same experimental set up as above. The reflected PL passed back through the microscope objective into a Princeton Instruments 0.3 m spectrometer and Picoquant PMT (with an instrument response function width of ~150 ps) connected to a Timeharp Picoquant time-correlated photon counting system. The dispersed PL was reflected towards a photomultiplier connected to a commercial photon counting system. Measurements of the lifetimes of the confined states were then carried out over a range of excitation power densities.

Thermogravimetric analysis (TGA):

TGA analysis was performed under a nitrogen atmosphere from room temperature to 1000 °C to evaluate the thermal stability of the material before and after optimization. The two samples show nearly identical behavior, except for sharper weight-loss steps in the optimized sample. The corresponding TGA curves are shown in Figure S4. Three distinct weight-loss events are observed: the first, around 290 °C, corresponds to an ~11.3% reduction, attributed to the removal of surface organic ligands; the second and third, at ~490 °C and ~670 °C, correspond to reductions of ~42.8% and ~44.5%, respectively, associated with decomposition of the perovskite framework.

Ligand Volume-Dependent Growth of $Cs_3Bi_2I_9$ NCs:

We studied the influence of varying the volume of the ligands (OA and OLm) on the size and shape of $Cs_3Bi_2I_9$ NCs while maintaining the optimized Cs_2CO_3:BiI_3 molar ratio of 1:2.70 (see Figure S1). Primarily, the colour of the $Cs_3Bi_2I_9$ NCs dispersed in hexane undergoes a noticeable transition from deep red to vibrant orange as the ligand volume increases from 0.3 mL to 0.8 mL (see Figure S16). This distinct colour shift clearly suggests that the possible variations in the size and morphology of the NCs. Furthermore, we observed a slight variation in the main feature peak from 500 nm to 485 nm as the ligand volume increased from 0.3 to

0.8 mL, indicating a minor change in the bandgap. However, due to the strong scattering tail, we are unable to estimate the precise bandgap value (see Figures S17c to S19c). The TEM analysis of NCs with a low ligand volume (0.3 mL) showed that the majority of the NCs exhibit a trigonal shape with self-assembly along with hexagonal NCs (see Figure S17). The scattering tail in the corresponding absorption spectra shows a strong and sharp increase in the low energy region (see in Figure S17c). As the ligand volume increases, however, the number of hexagonal-shaped NCs also rises with a greater improvement in uniformity of the size and a reduction in the scattering tail of their absorption spectra. As shown in the previous section (Figure 3b), monodisperse, nearly hexagonal-shaped $Cs_3Bi_2I_9$ NCs were obtained at an optimal ligand volume of 0.5 mL, with a negligible scattering tail in the absorption spectra (see Figure 3e). When the ligand volume exceeds this optimal value, the size variation of the hexagonal NCs begins to increase (similar to the BiI_3 concentration-dependent study in the previous section), along with a small number of trigonal-shaped NCs (see Figure S18). This size variation becomes more pronounced with further increases in ligand volume, resulting in the exclusive presence of both larger and smaller NCs (see in Figure S19). Likewise, the scattering tail in the UV-visible absorption spectra emerges in the low-energy region and becomes more pronounced as the ligand volume is further increased (see Figures S17c and S19c). The growth of NCs under varying ligands volume likely follows a mechanism similar to that of precursor concentration-dependent NCs growth, as explained in the main text. At lower ligands volume, the growth tends to be anisotropic, favours the formation of elongated or irregularly shaped NCs (Figure S17). As the ligands volume increases, it gradually stabilizes the NCs surface, promoting more uniform growth. When the ligands volume reaches its optimal level of 0.5 mL, it facilitates well-controlled growth leading to the formation of uniformly shaped hexagonal NCs (Figure 3b). However, beyond this optimal volume, an excessive ligand-rich environment

may trigger a ripening process, where larger NCs grow at the expense of smaller ones, potentially altering the overall size distribution.

Time-controlled synthesis of $Cs_3Bi_2I_9$ NCs:

To further understand the growth process of the NCs, we studied the time-controlled reaction at 2, 4, 6, 8, 10, and 12 minutes under optimized synthesis conditions (Cs_2CO_3:BiI_3 = 1:2.70; 0.5 mL each of OA and OLm) by monitoring TEM images and UV–vis absorption spectra, as shown in Figures S20 and S21. The detailed synthesis procedure is provided in the Supporting Information. At the early stage of the reaction (2 minutes), precursor particles appeared to aggregate, forming large agglomerates along with a few smaller particles (Figure S20a), indicating the initiation of the reaction between the precursors and the beginning of nucleation. By 3 minutes, well-separated tiny particles became clearly visible, exhibiting distinct contrast variations in the TEM images. The brighter regions were typically concentrated in the central area, suggesting that nucleation of NCs was in process, possibly due to the accumulation of high-Z metal atoms (i.e., Bi) in the core region of the particles, although no well-defined shapes are observed at this stage. By 4 minutes, well-defined square-like particles began to form, with reduced contrast variation and these square-like particles transformed into trigonal-shaped NCs started to appear alongside a few hexagonal-shaped NCs at 6 minutes. As the reaction continued to 8, 10, and 12 minutes, the proportion of trigonal-shaped NCs decreased, and well-defined, nearly hexagonal-shaped NCs became more prominent, indicating progressive shape evolution and improved crystallinity over time. Similarly, UV–vis absorption spectra at the early stage (2 minutes) exhibited a broad peak centered at 490 nm, along with sub-bands at 420 and 338 nm (Figure S21), indicating the instantaneous formation of isolated $[Bi_2I_9]^{3-}$ clusters. However, as the reaction time increased from 3 to 12 minutes, the broad bands became progressively sharper with a more defined absorption edge, consistent with the morphological transformation of tiny particles into square-like, then trigonal, and eventually well-defined

hexagonal-shaped nanocrystals. It is worth mentioning that our synthetic strategy facilitates the rapid and controlled formation of $Cs_3Bi_2I_9$ NCs with well-defined morphology and nearly uniform size distribution, demonstrating a significant advancement over conventional methods, which typically require longer reaction times and often yield products with less uniformity in both shape and size.

Transient absorption (TA) measurement

TA measurements were performed on a HARPIA-TA system (Light Conversion). A Yb:KGW laser (PHAROS, 1035 nm, 10 kHz, pulse width 163 fs, Light Conversion) was divided into two beams. One beam is directed into an optical parametric amplifier (ORPHEUS-NEO) to generate the pump at 400 nm. The other beam is focused onto a 5 mm sapphire crystal to generate a low-intensity continuum probe. The magic angle of 54.7° was set the polarization of the pump and probe beams. A mechanical delay stage varied the time delays between the pump and the probe beams. The pump and probe beams were spatially overlapped on the sample, and the transmitted probe was collected using an Andor Kymera 193i spectrograph.

Optical-pump-terahertz-probe (OPTP) measurements

The terahertzprobe spectrometer based on an ultrafast Ti:sapphire amplifier (Newport Spectra Physics Spitfire Ace, 13 mJ, 1 kHz, 40 fs) is used for OPTP measurements. An optical parametric amplifier (TOPAS Prime, Light Conversion), pumped by 3 mJ of the amplifier's beam, generated 400 nm pump pulses with a time duration of 50 fs. The terahertz probe was generated by optical rectification in GaP and detected by electro-optic sampling in ZnTe using a balanced photodiode scheme and a high-resolution oscilloscope (Pico Technology PicoScope 4262) for data acquisition. A four pulse scheme, with the terahertz beam chopped at 500 Hz and the optical pump chopped at 250 Hz, provided the transmitted terahertz electric field amplitudes E_{off} and E_{on} , which could be used to calculate $\Delta T/T = (E_{on} - E_{off})/ E_{off}$.

Fabrication and characterization of the X-ray detector

The bulk scale synthesized colloidal $Cs_3Bi_2I_9$ NCs were centrifuged at 10000 rpm for 10 minutes. The resulting precipitate was collected and vacuum-dried overnight. The dried material was ground using a ceramic mortar and pestle to obtain $Cs_3Bi_2I_9$ NCs fine powder. 400 mg of this NC powder was pressed into pellets using a 10 mm pellet press die under a pressure of 156 MPa with a hydraulic press. The resulting pellets had a thickness of 1.4 mm and a shiny surface. A thick film of $Cs_3Bi_2I_9$ NCs was prepared by drop-casting 100 µL of the $Cs_3Bi_2I_9$ NC solution onto pre-cleaned, patterned FTO-coated glass substrates preheated to 60 °C on a hot plate. SEM images of the pellet and thick film confirm uniform nearly pinhole-free morphology (Figure S29). Circular Au electrodes, each with a diameter of 5 mm and a thickness of 80 nm, were deposited on both sides of the pellet using a metal shadow mask and a vacuum thermal evaporation system. For the thick film, six electrode pixels, each with an area of 0.25 cm^2, were deposited using the same procedure. The Au/ $Cs_3Bi_2I_9$ NCs pellet/Au device was affixed to a glass slide using silver paste and epoxy glue. Fine wires were connected to the top and bottom electrodes, with copper pads serving as contact points for measurements. X-ray photo I-V characteristics and dynamic X-ray current response measurements were performed using a Keithley 2470 sourcemeter tungsten (W) X-ray source operating at a tube voltage of 60 kV and varying power to control the dose rates. Figure S30 shows the simulated X-ray spectrum of a tungsten X-ray source operating at a tube voltage of 60 kV, with an aluminium attenuator. The dose rates of the X-ray set were measured using Radcal ADDM+ ion chamber dose meter.We irradiated the entire thick film device for X-ray photocurrent measurements. However, given the vertical structure of our devices (FTO/$Cs_3Bi_2I_9$ thick film/Au), we expect only a minimal contribution to the photocurrent from regions outside the active area defined by the electrodes.

Calculation of mobility-lifetime (μτ) using Hecht fitting

Figure S20(a) shows the photo I-V characteristics of the device fitted using a conventional Hecht equation as follows.

$$I = \frac{I_0 \mu \tau V}{L^2}(1 - \exp\left(-\frac{L^2}{\mu \tau V}\right)) \quad (1)$$

Here, I represents the photocurrent, I_0 is the saturated photocurrent, L denotes the thickness of the active material, V is the applied voltage,

Figure S20(b) shows the photo I-V characteristics of the device fitted using a modified Hecht equation as follows.

$$I = \frac{I_0 \mu \tau V}{L^2}\frac{(1 - \exp\left(-\frac{L^2}{\mu \tau V}\right))}{1 + \frac{L}{V}\frac{s}{\mu}} \quad (2)$$

Where s is the surface recombination velocity. The μτ values are obtained to be 3.6×10^{-4} cm^2 V^{-1} and 2.4×10^{-4} cm^2 V^{-1} using conventional and modified Hecht fit, respectively.

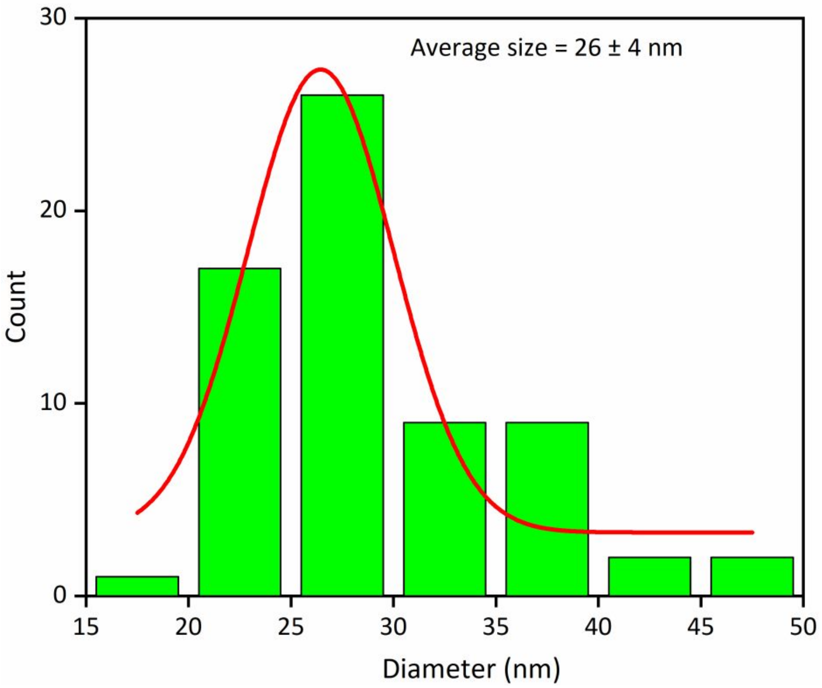

Figure S1. The size distribution analysis of $Cs_3Bi_2I_9$ NCs derived from particle counts obtained through HR-STEM imaging.

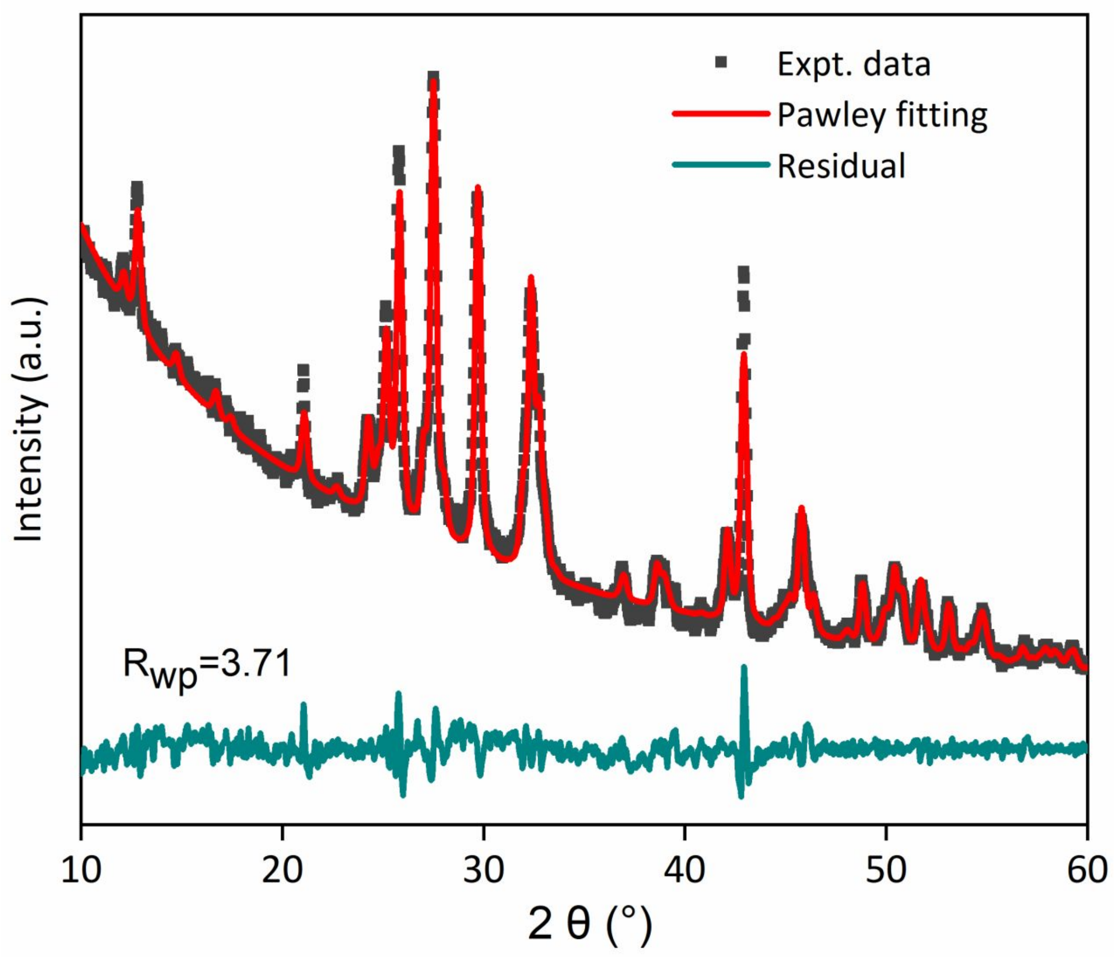

Figure S2. Pawley refinement of the X-ray diffraction data of the NCs, with a low residual (Rwp = 3.7%), demonstrating high crystallinity and phase purity.

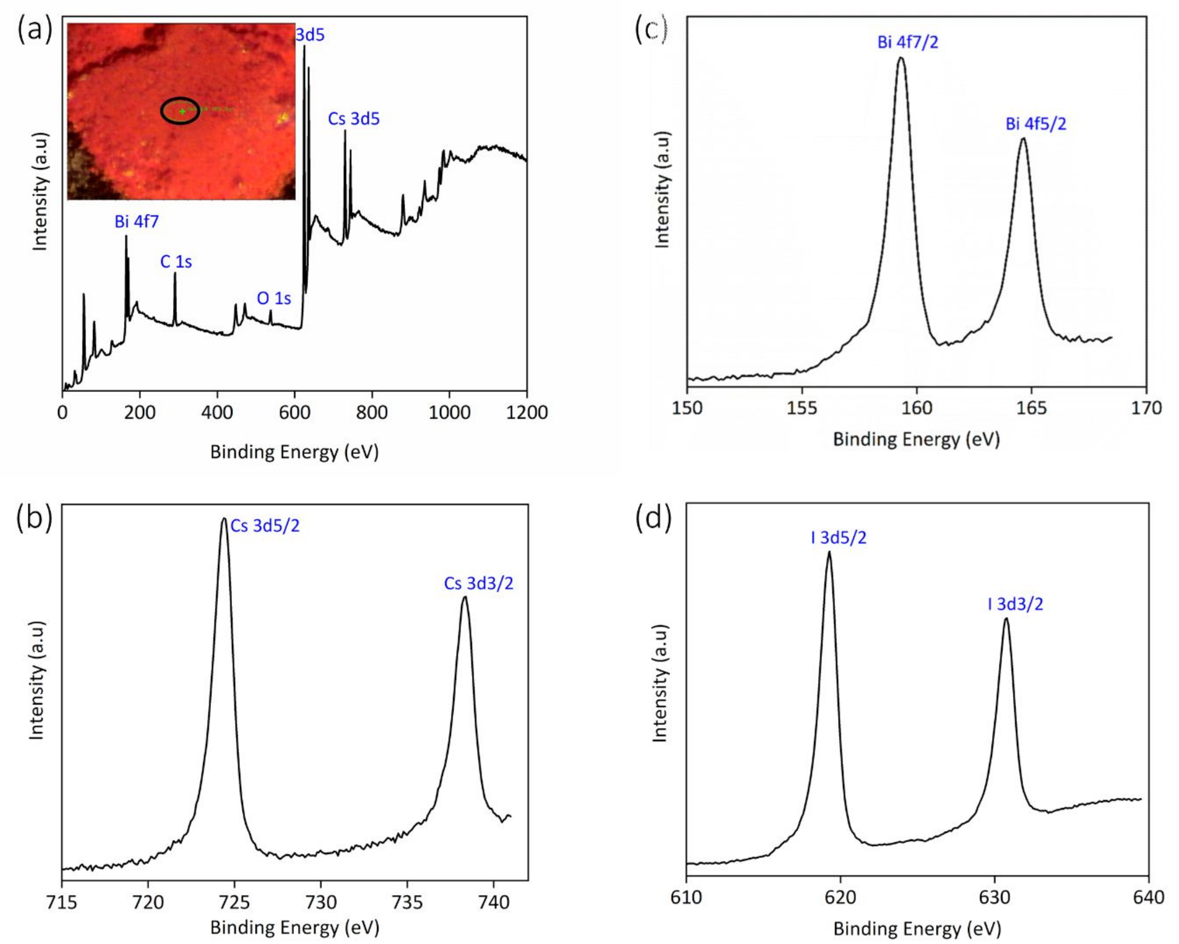

Figure S3. X-ray photoelectron spectra of $Cs_3Bi_2I_9$ NCs. (a) Survey spectrum, with the inset showing the sample image highlighting the position from which the spectra were collected; (b) high-resolution Cs 3d spectrum; (c) high-resolution Bi 4f spectrum; and (d) high-resolution I 3d spectrum.

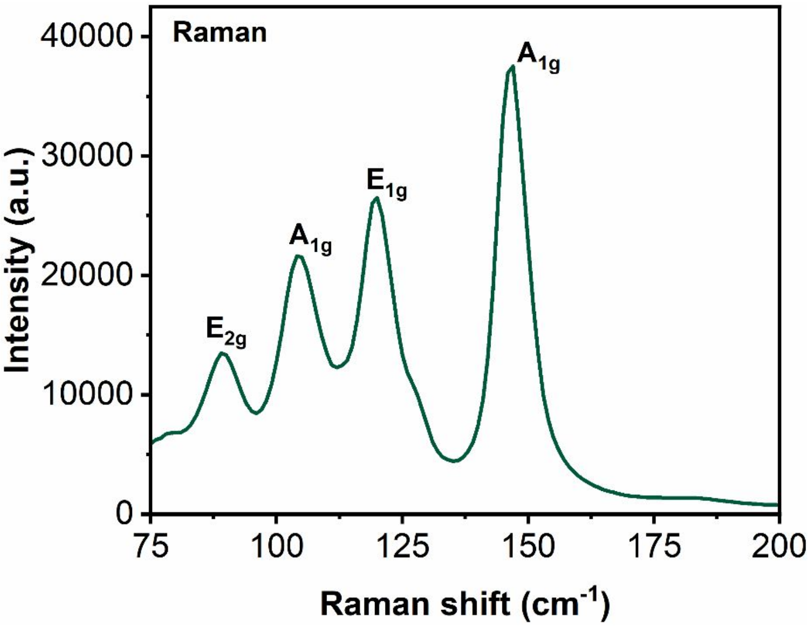

Figure S4. Raman spectra of $Cs_3Bi_2I_9$ NCs film.

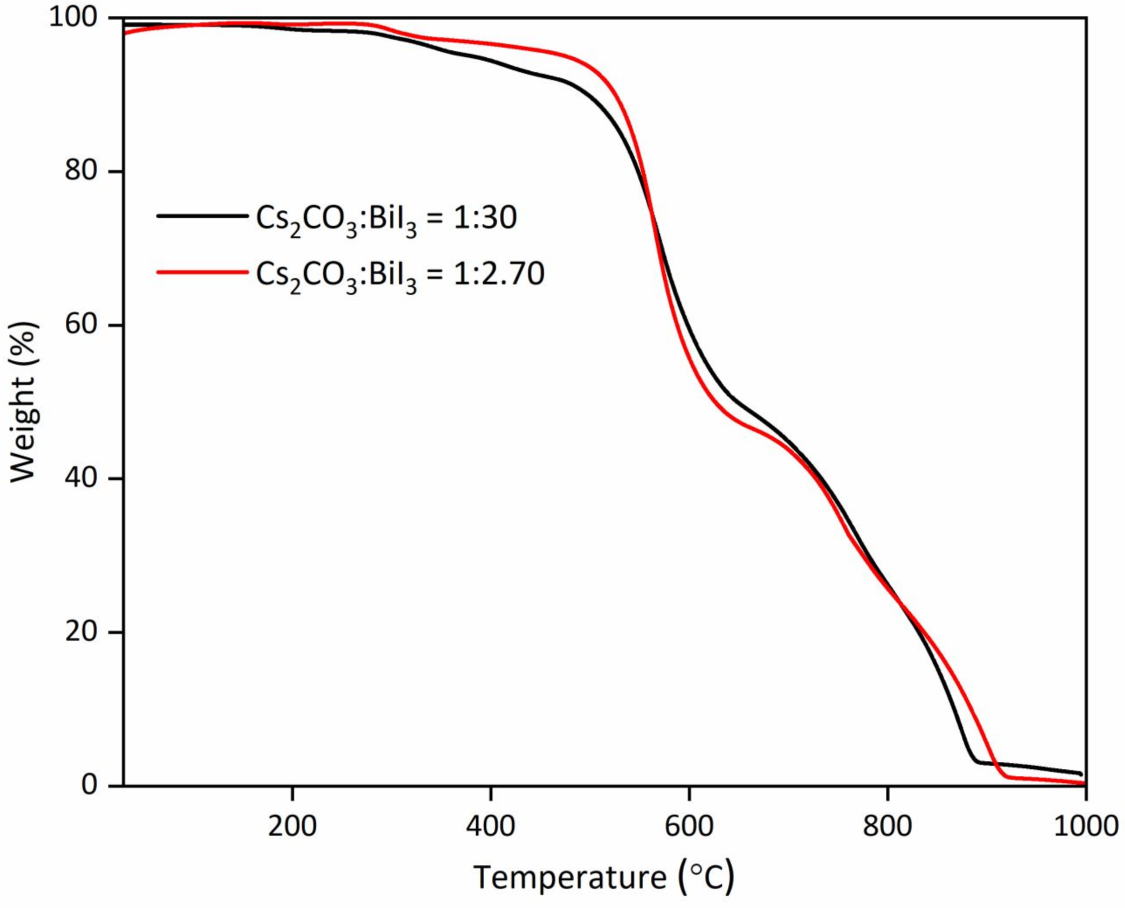

Figure S5. Thermogravimetric analysis (TGA) of $Cs_3Bi_2I_9$ NC samples before and after optimization. Color code: red, after optimization (Cs_2CO_3:BiI_3 = 1:2.70); black, before optimization (Cs_2CO_3:BiI_3 = 1:3).

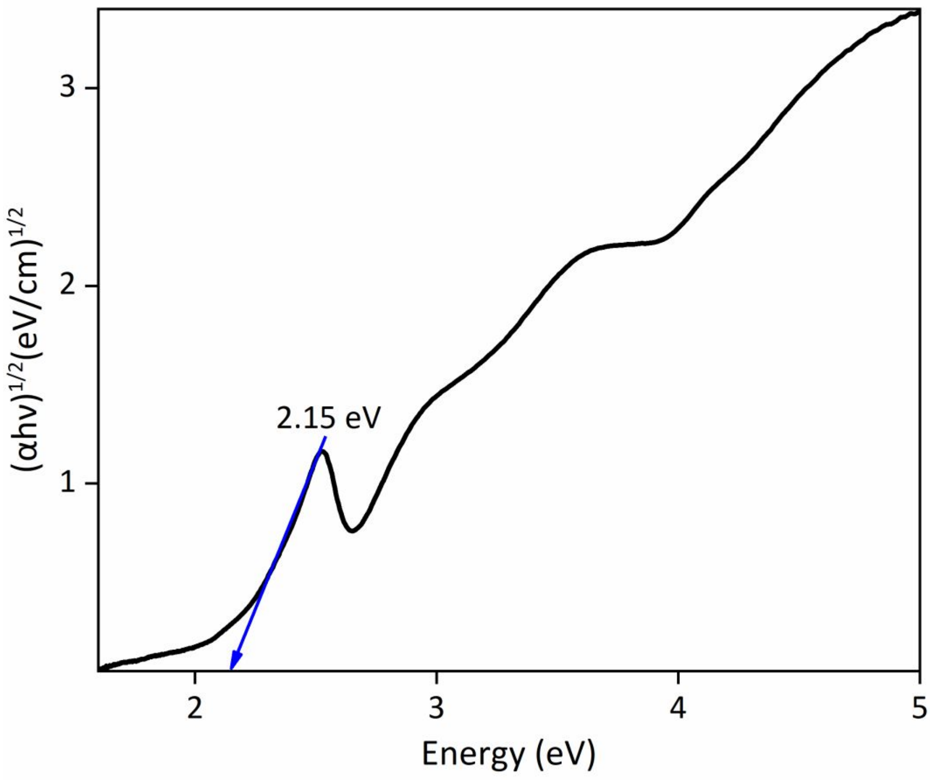

Figure S6. Tauc plot for indirect band gap estimation of $Cs_3Bi_2I_9$ NCs derived from their UV-Visible absorption spectra. The plot of $(\alpha h\nu)^{1}$□^{2} versus photon energy ($h\nu$) was used to extrapolate the band gap value.

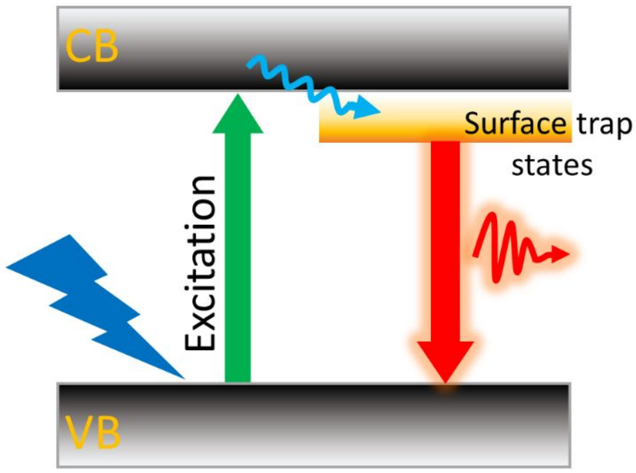

Figure S7. Proposed mechanism for radiative recombination in $Cs_3Bi_2I_9$ NCs at longer wavelengths.

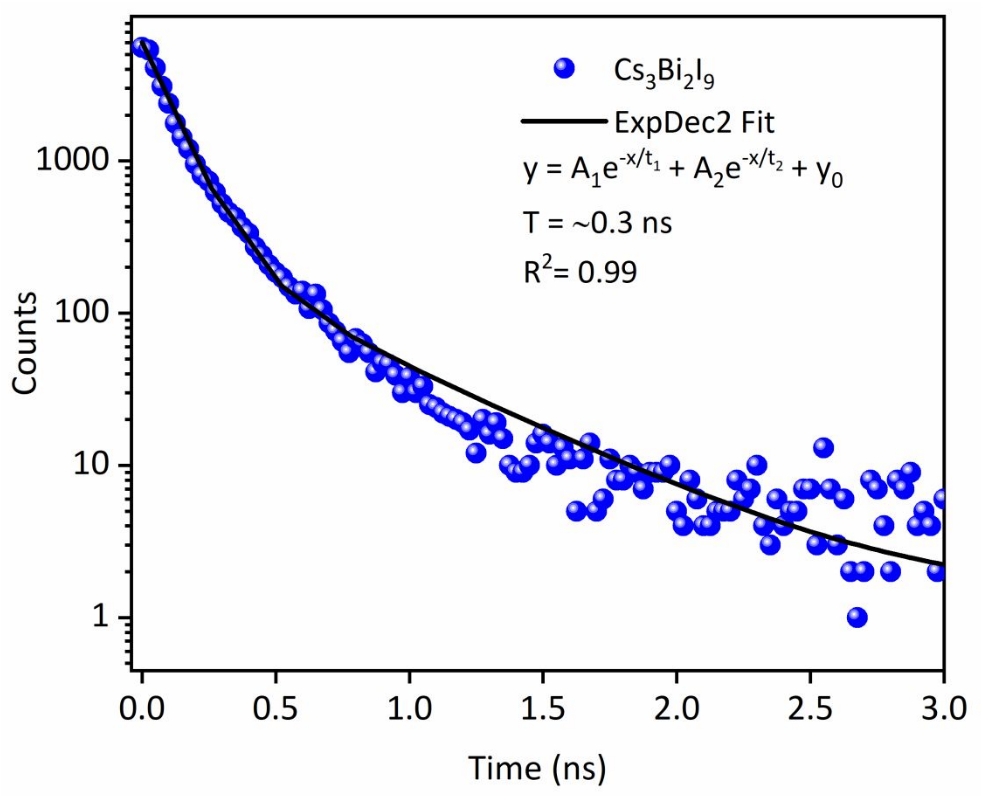

Figure S8. PL decay dynamics spectra, along with a biexponential decay fit line.

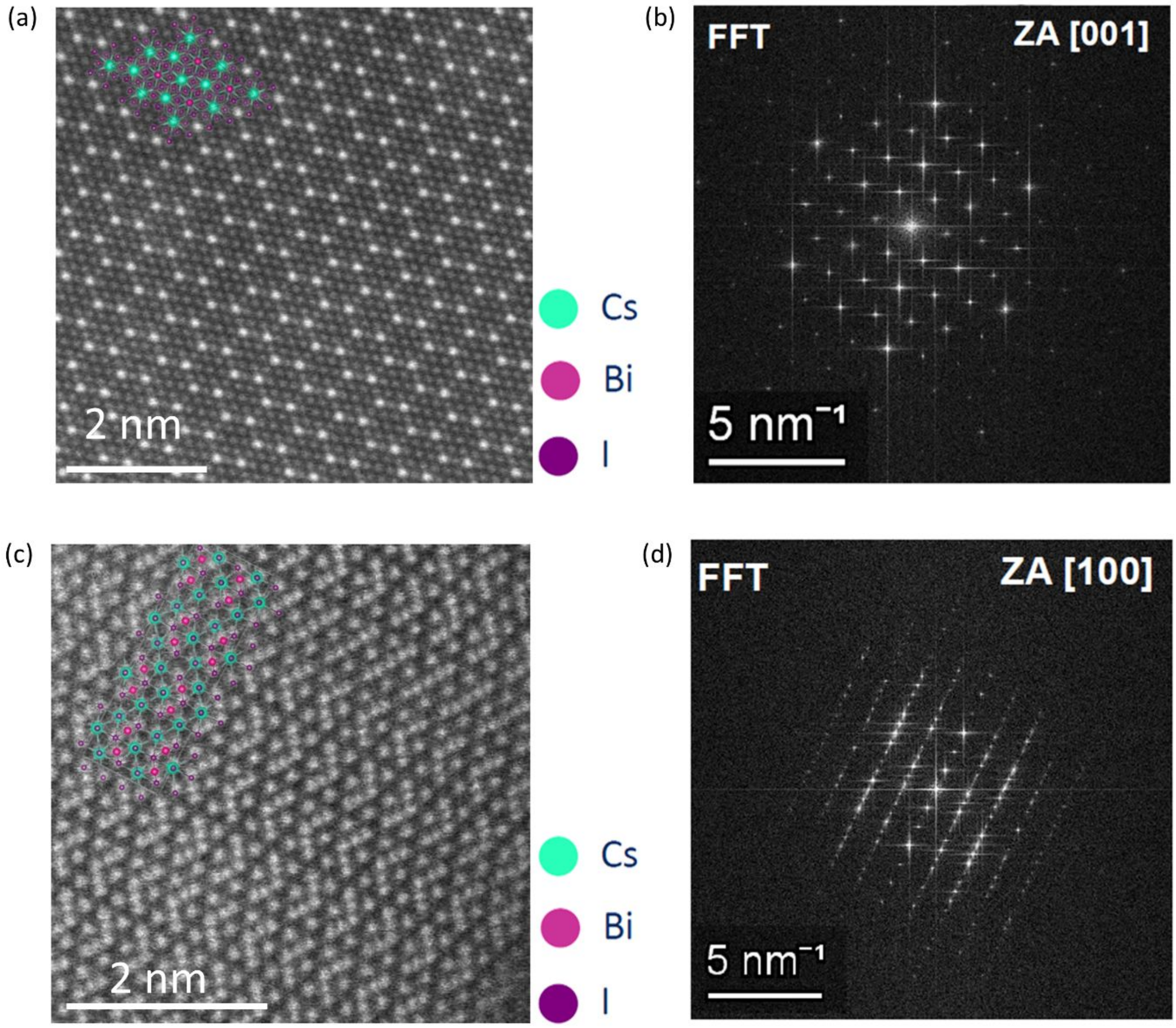

Figure S9. (a) and (c) High-resolution STEM images of $Cs_3Bi_2I_9$ NCs along the [001] zone axis in (a) and the [100] zones in (c). The overlay illustrates the atomic structure of $Cs_3Bi_2I_9$ in these orientation. (b) and (d) Corresponding FFT patterns confirming the hexagonal phase of $Cs_3Bi_2I_9$ along the [001] and [100] zone axes, respectively.

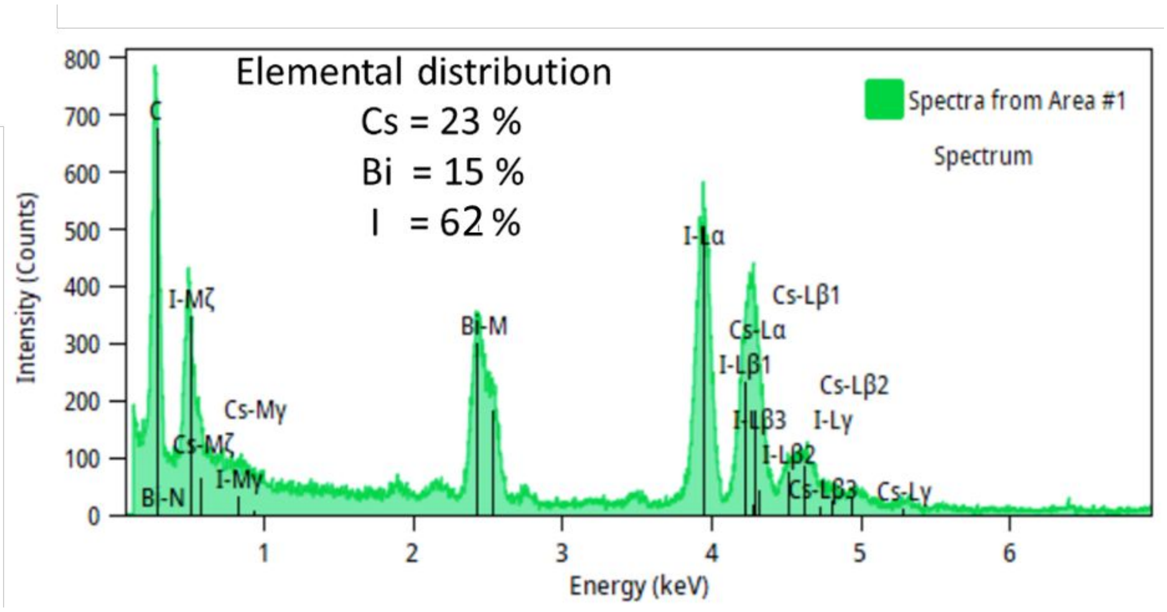

Figure S10. EDX spectrum of the $Cs_3Bi_2I_9$ NCs shown in Figure 2(d).The quantitative elemental analysis of the EDX spectrum confirming the stoichiometric distribution of Cs, Bi, and I within the $Cs_3Bi_2I_9$ NCs.

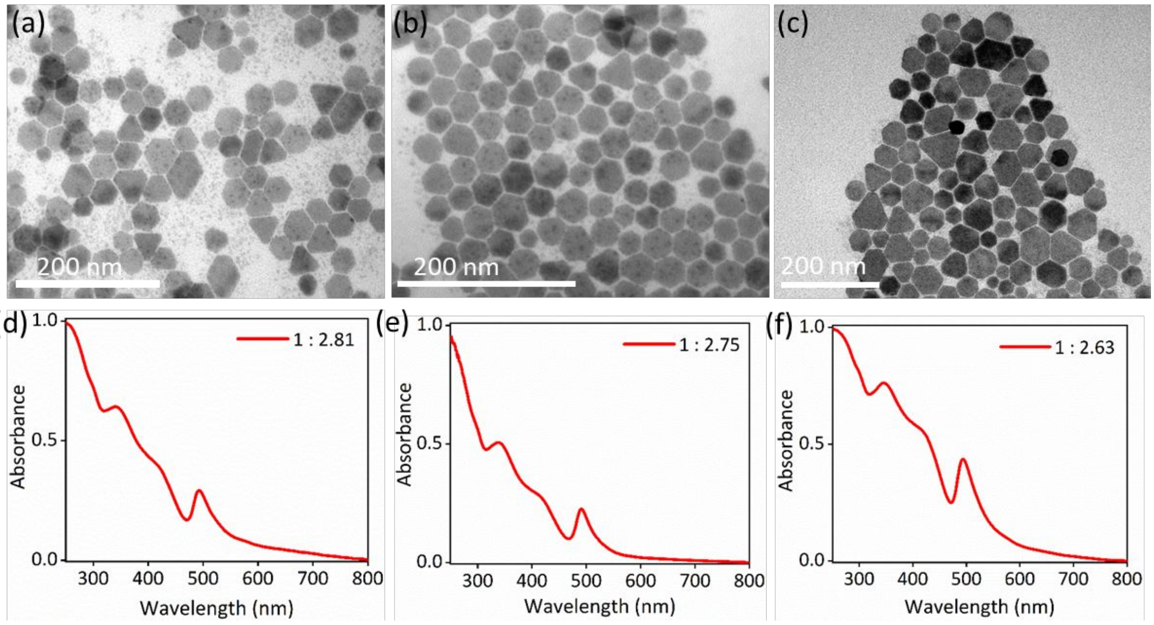

Figure S11. Optimization of $Cs_3Bi_2I_9$ NCs by varying the BiI_3 molar ratio, along with their corresponding UV-Vis spectra and the proposed mechanism. (a) TEM image of $Cs_3Bi_2I_9$ NCs synthesized with a Cs_2CO_3:BiI_3 molar ratio of 1:2.81 and (d) the corresponding UV-Vis spectra, showing reduced light scattering as the BiI_3 ratio decreases. (b) TEM image of $Cs_3Bi_2I_9$ NCs synthesized with a Cs_2CO_3:BiI_3 molar ratio of 1:2.75 and (e) the corresponding UV-Vis spectra, indicating negligible light scattering at lower BiI_3 ratios. (c) TEM image of $Cs_3Bi_2I_9$ NCs synthesized with a Cs_2CO_3:BiI_3 molar ratio of 1:2.63 and (f) the corresponding UV-Vis spectra, suggesting increased light scattering when the BiI_3 ratio is further reduced.

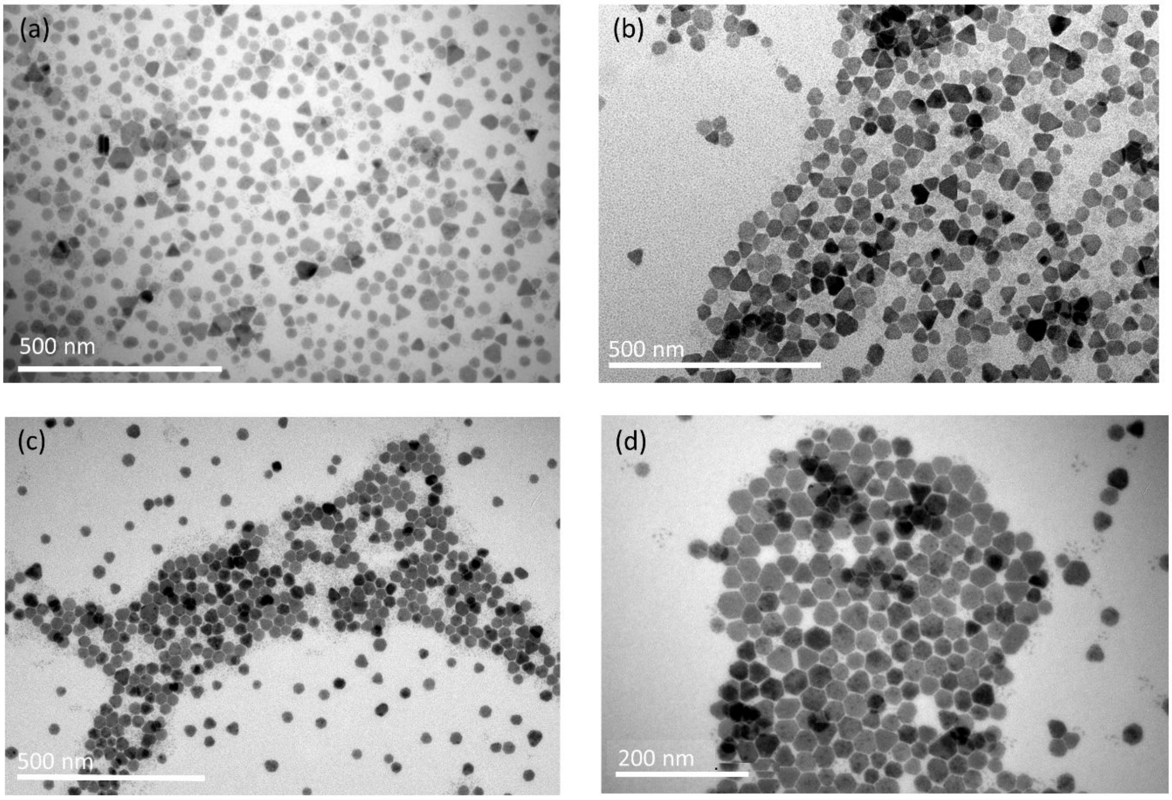
(a)
500 nm
(b)
500 nm
(c)
500 nm
(d)
200 nm

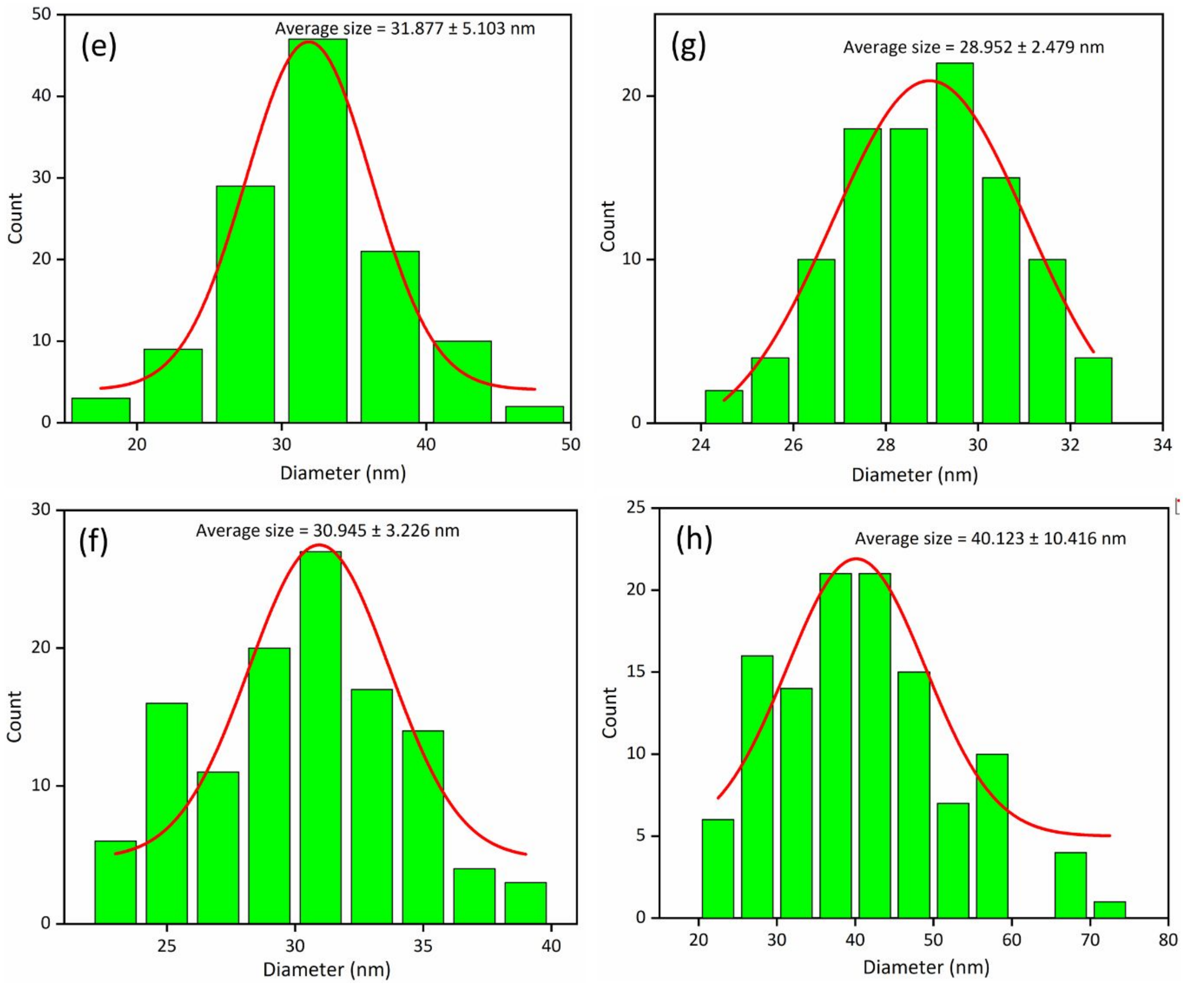

Figure S12. Optimization of $Cs_3Bi_2I_9$ NCs by varying the BiI_3 molar ratio. (a) TEM image of $Cs_3Bi_2I_9$ NCs synthesized with a Cs_2CO_3:BiI_3 molar ratio of 1:3. (b) TEM image of $Cs_3Bi_2I_9$ NCs synthesized with a Cs_2CO_3:BiI_3 molar ratio of 1:2.81. (c) TEM image of $Cs_3Bi_2I_9$ NCs synthesized with a Cs:BiI_3 molar ratio of 1:2.75. (d) TEM image of $Cs_3Bi_2I_9$ NCs synthesized with a Cs_2CO_3:BiI_3 molar ratio of 1:2.70. (e) Size distribution analysis of $Cs_3Bi_2I_9$ NCs synthesized with a Cs:BiI_3 molar ratio of 1:2.81 derived from particle measurements obtained through TEM imaging. (f) Size distribution analysis of $Cs_3Bi_2I_9$ NCs synthesized with a Cs:BiI_3 molar ratio of 1:2.75 derived from particle measurements obtained through TEM imaging. (g) Size distribution analysis of $Cs_3Bi_2I_9$ NCs synthesized with a Cs:BiI_3 molar ratio of 1:2.70 derived from particle measurements obtained through TEM imaging. (h) Size distribution analysis of $Cs_3Bi_2I_9$ NCs synthesized with a Cs:BiI_3 molar ratio of 1:2.63 derived from particle measurements obtained through TEM imaging.

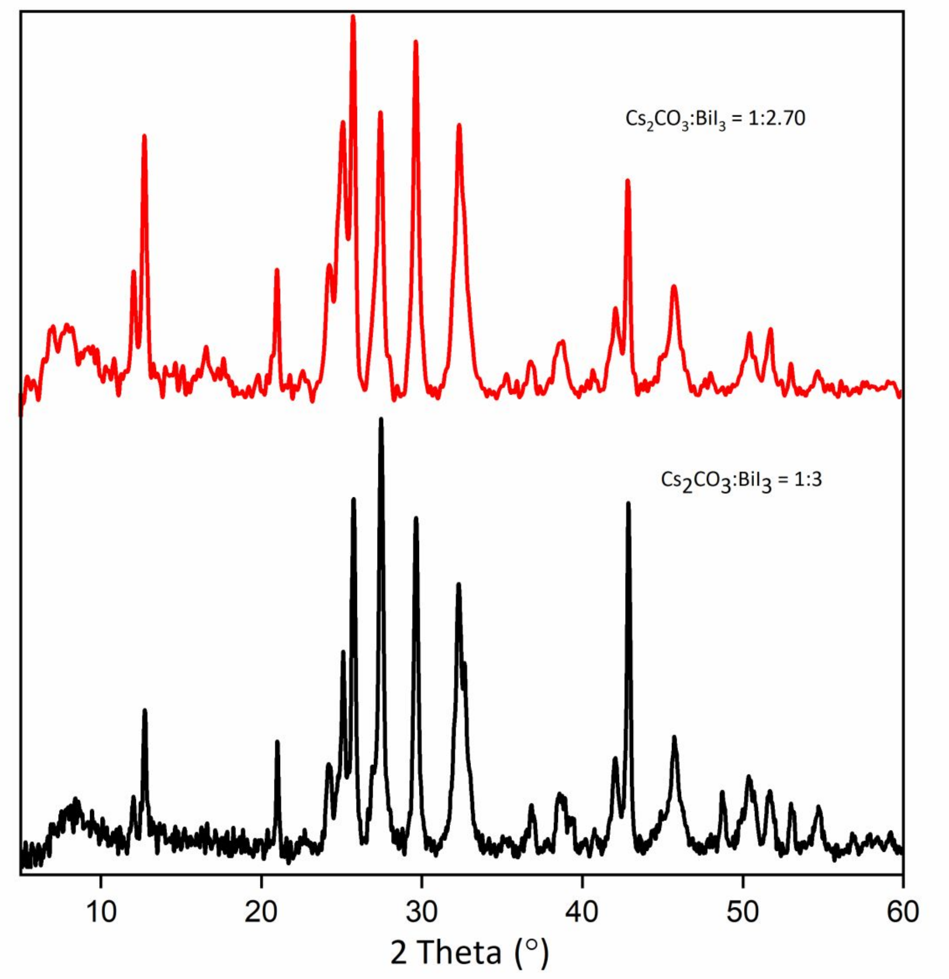

Figure S13. XRD patterns of $Cs_3Bi_2I_9$ NCs before and after optimization. Color code: red, after optimization; black, before optimization.

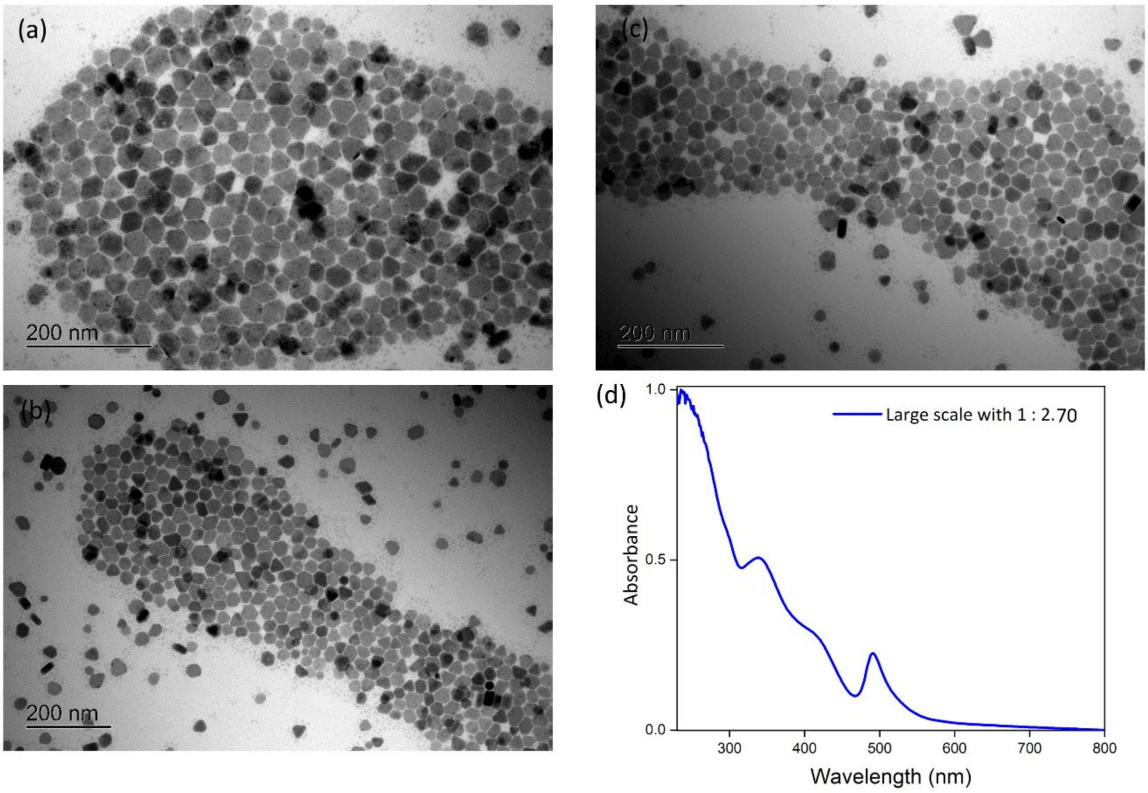

Figure S14. (a–c) TEM images of $Cs_3Bi_2I_9$ NCs synthesized on a large scale using an optimized Cs:BiI_3 molar ratio of 1:2.70, and (d) the corresponding UV-Visible absorption spectra.

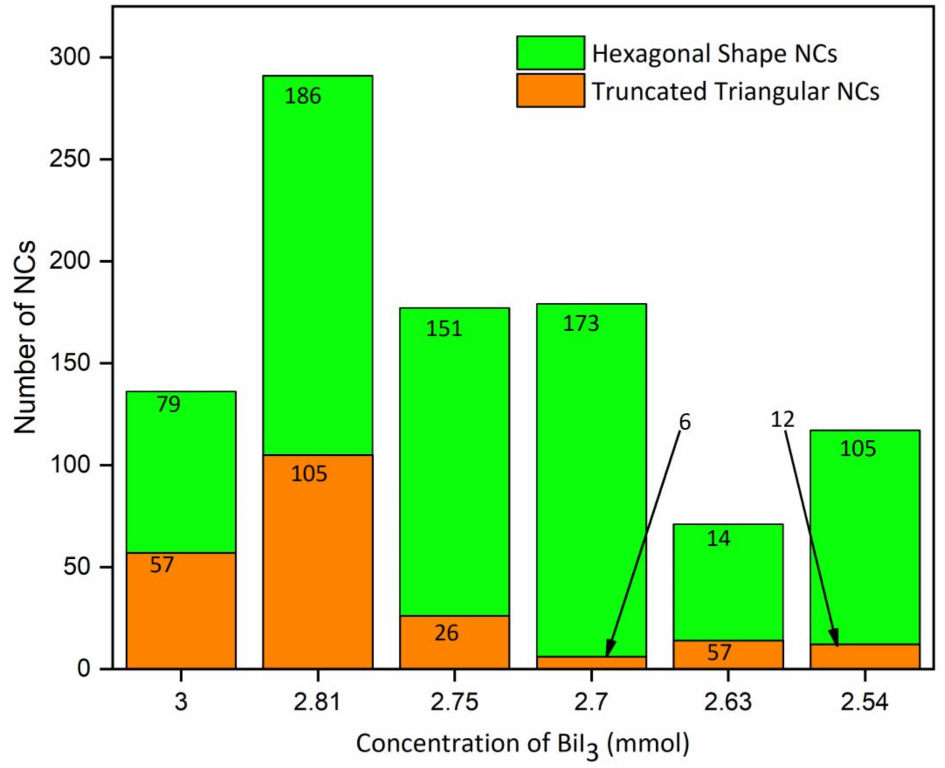

Figure S15. Statistical estimation of $Cs_3Bi_2I_9$ NCs under varying concentrations of BiI_3.

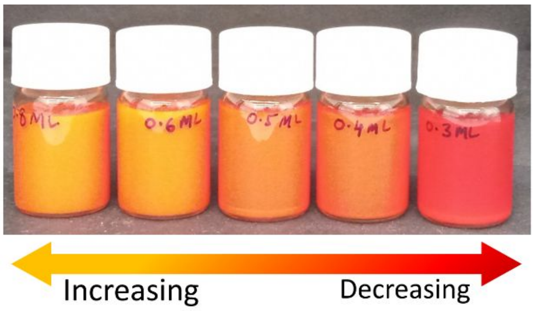

Figure S16. Digital images of $Cs_3Bi_2I_9$ NCs (dispersed in hexane) synthesized with varying ligand volumes, ranging from 0.8 mL (left) to 0.3 mL (right).

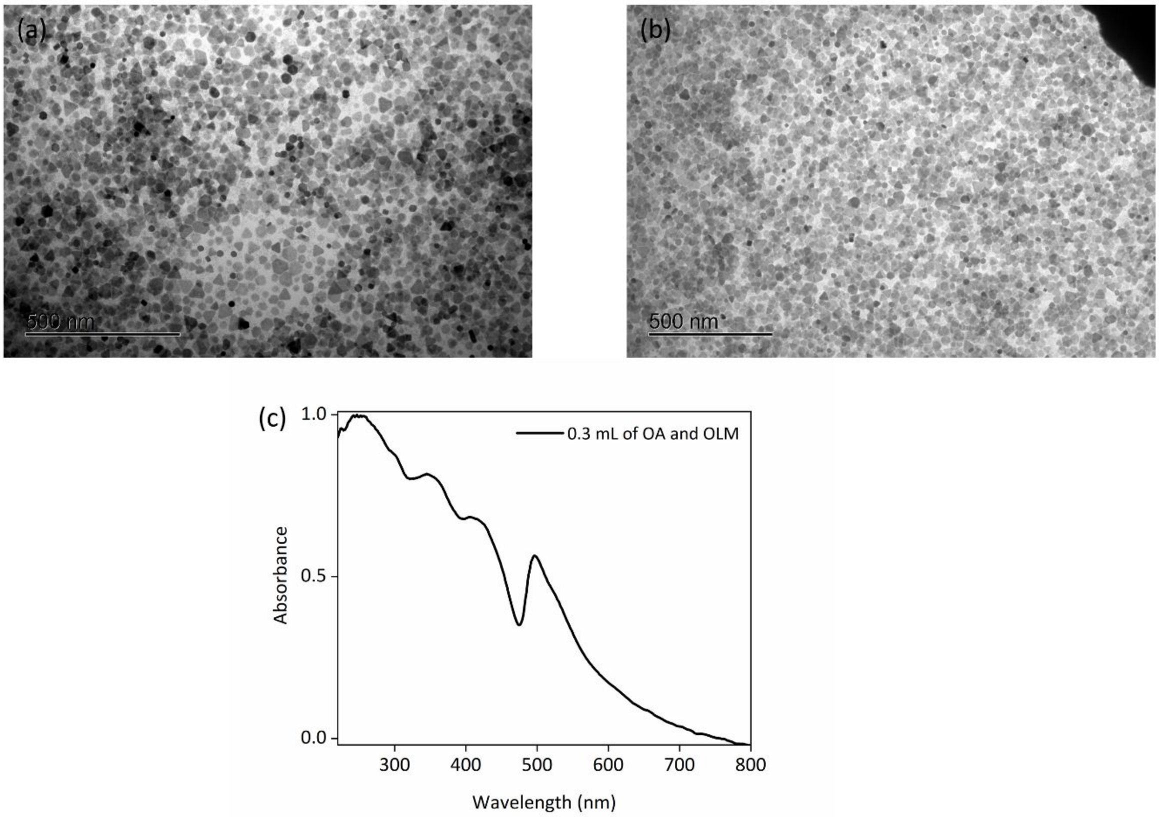

Figure S17. (a, b) TEM images of $Cs_3Bi_2I_9$ NCs synthesized using an optimized Cs:BiI_3 molar ratio of 1:2.70 with a low ligand volume (0.3 mL), and (c) the corresponding UV-Visible absorption spectra.

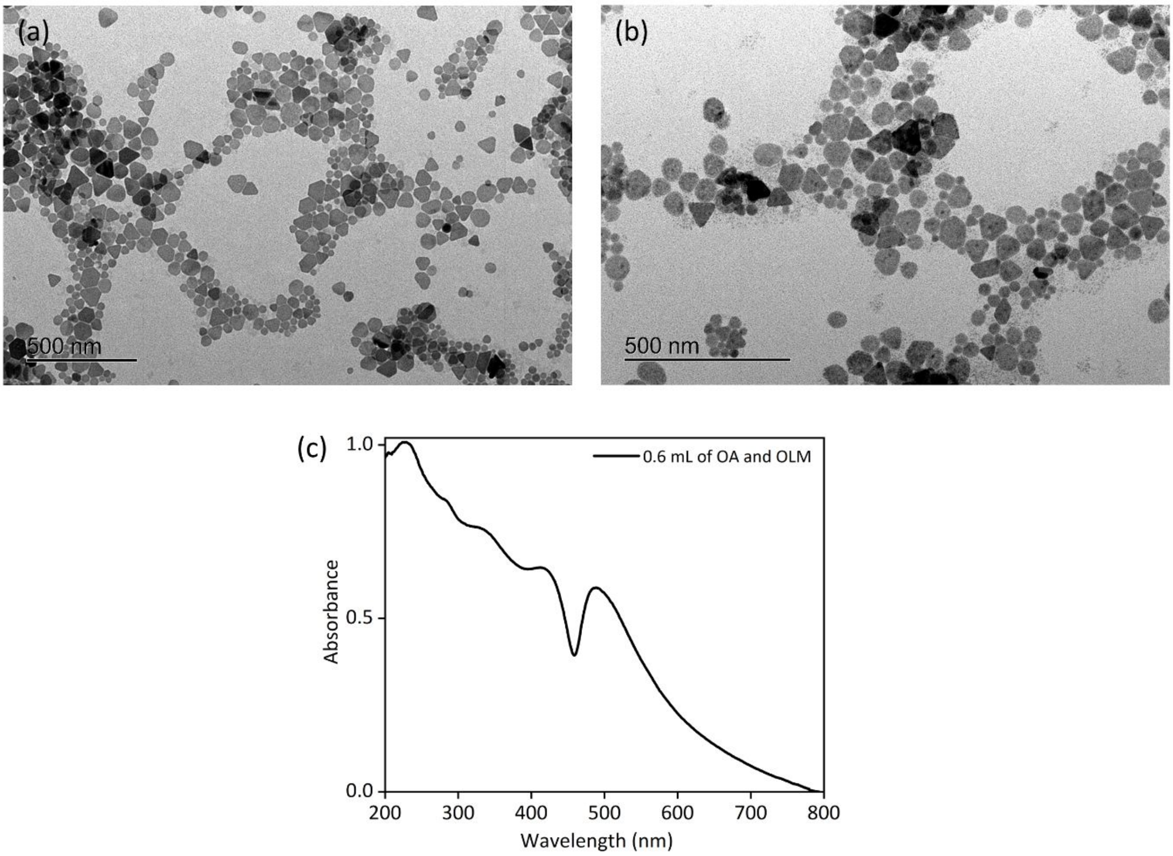

Figure S18. (a, b) TEM images of $Cs_3Bi_2I_9$ NCs synthesized using an optimized Cs:BiI_3 molar ratio of 1:2.70 with a ligand volume of 0.6 mL, and (c) the corresponding UV-Visible absorption spectra.

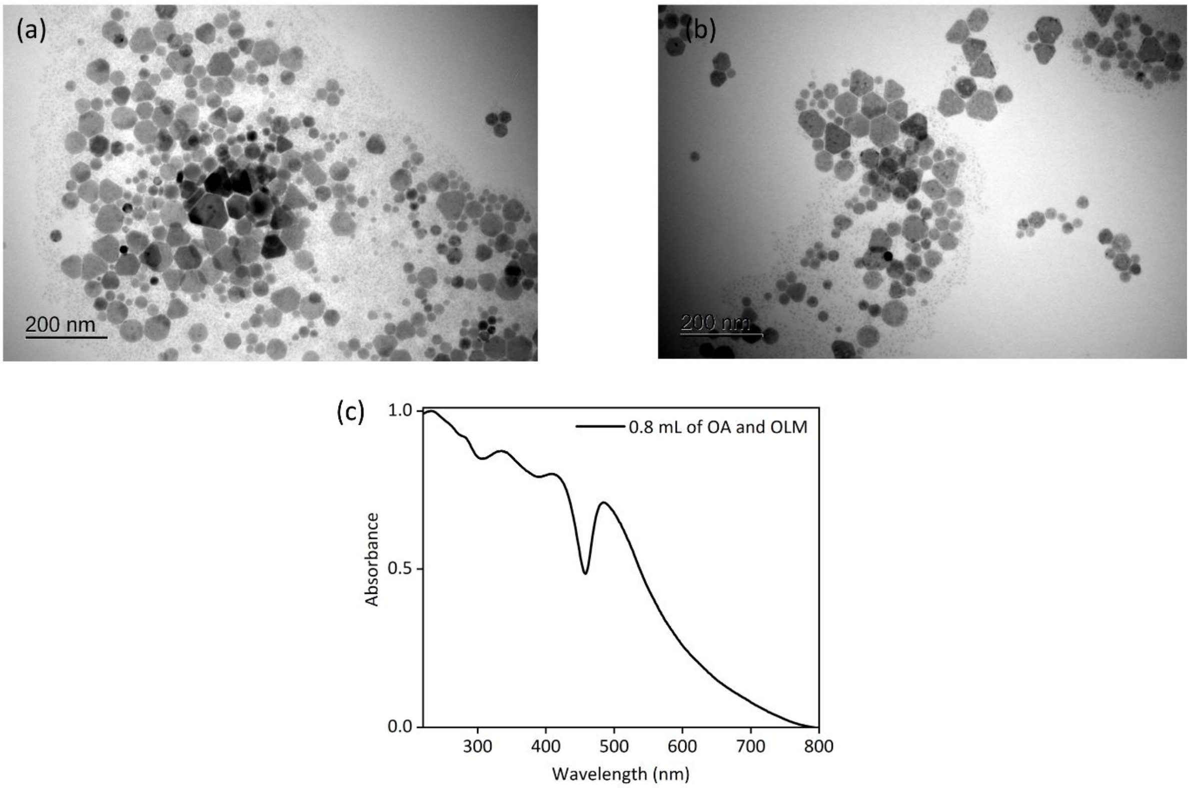

Figure S19. (a, b) TEM images of $Cs_3Bi_2I_9$ NCs synthesized using an optimized Cs:BiI_3 molar ratio of 1:2.70 with a ligand volume of 0.8 mL, and (c) the corresponding UV-Visible absorption spectra.

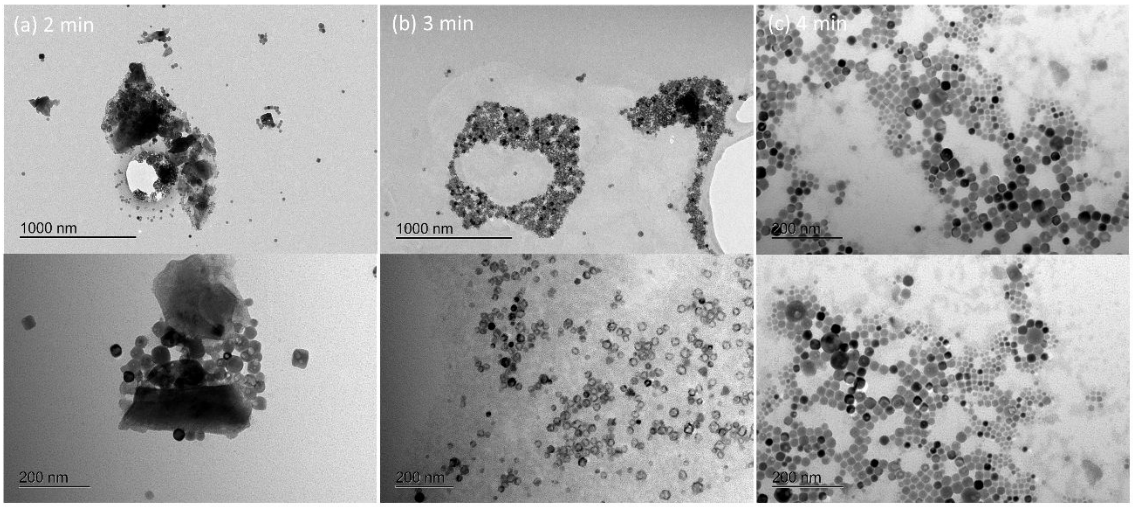

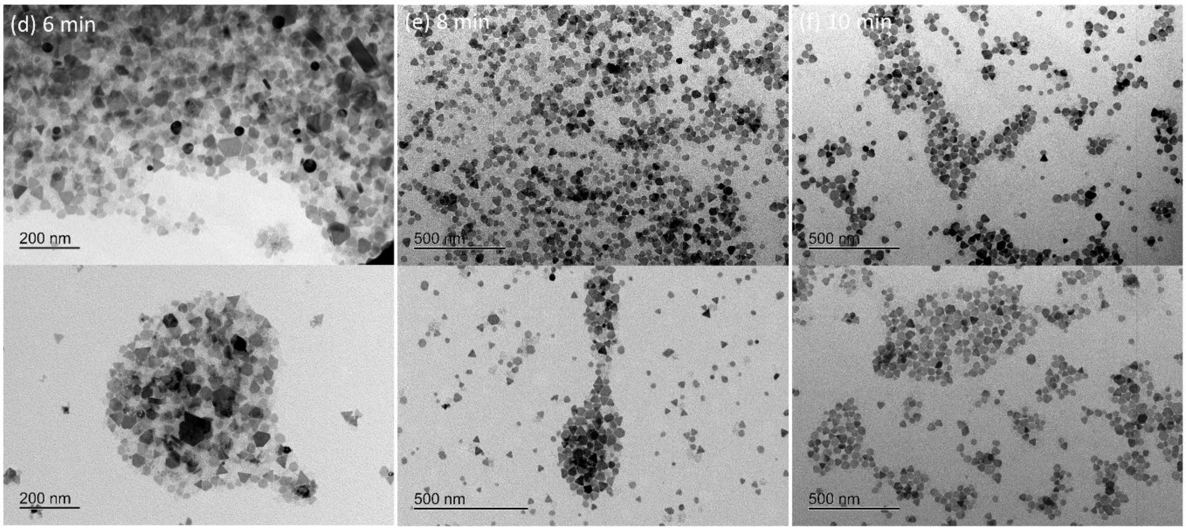

Figure S20. Time-controlled TEM images showing the morphological evolution of $Cs_3Bi_2I_9$ NCs at various reaction durations: (a) 2 min, (b) 3 min, (c) 4 min, (d) 6 min, (e) 8 min, and (f) 10 min. At 2 minutes, large agglomerates and few small particles are observed, suggesting early-stage precursor interaction. Well-separated small seed particles appear at 3 minutes, followed by the emergence of square-like NCs at 4 minutes. As the reaction continued, trigonal and hexagonal shapes begin to form 10 minutes, indicating a gradual shape evolution and improved crystallinity over time.

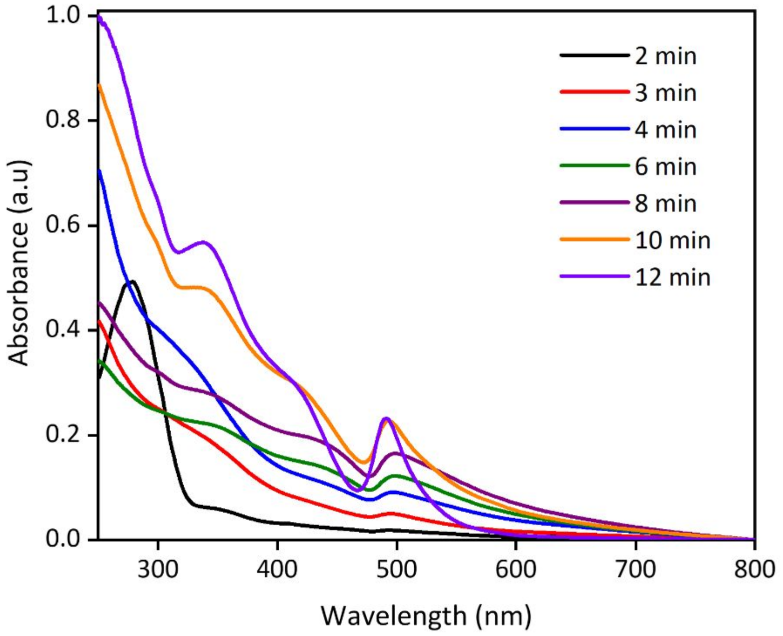

Figure S21. Time-dependent UV–vis absorption spectra of $Cs_3Bi_2I_9$ NCs recorded at different reaction intervals (2, 3, 4, 6, 8, 10, and 12 minutes) under optimized synthesis conditions (Cs_2CO_3:BiI_3 = 1:2.70; 0.5 mL each of OA and OLm). At the early stage (2 minutes), the spectrum displays a broad peak centered at 490 nm along with sub-bands at 420 and 338 nm, signifying the instantaneous formation of isolated $[Bi_2I_9]^{3-}$ clusters. With increasing reaction time, the absorption features gradually sharpen and the band edge becomes more distinct, accompanied by a notable reduction in the low-energy scattering tail. This spectral studies correlates with the morphological

transformation of the NCs from initial tiny particles to well-defined hexagonal-shaped nanocrystals, highlighting the progressive development of crystallinity and structural order.

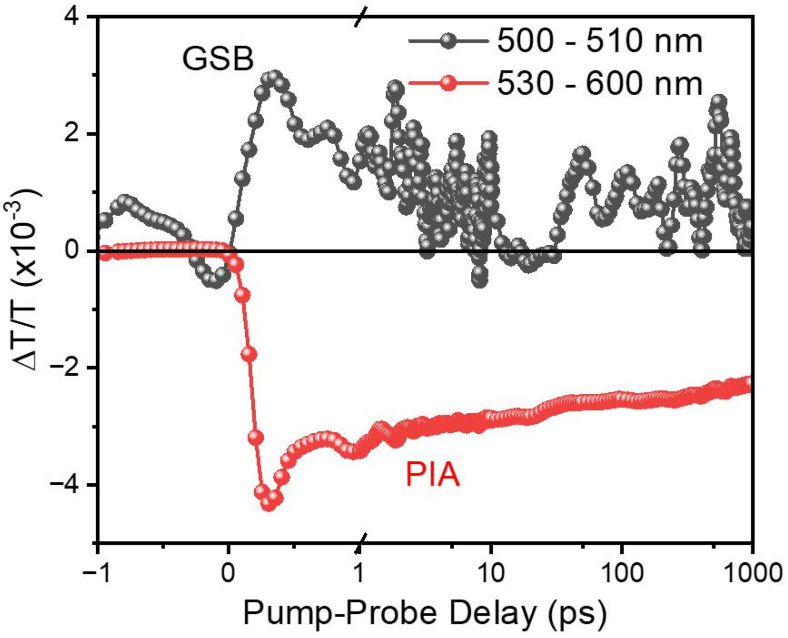

Figure S22. Comparison of ground-state bleach (GSB) and photo-induced absorption (PIA). Owing to the strong scattering light intensity from the fundamental pump laser (1030 nm wavelength, 1.20 eV), it is very challenging to resolve the GSB signal close to 2.5 eV in our setup. Nevertheless, the GSB kinetics could be still estimated to persist for over 1 ns, which is consistent with the result reported in the literature.[1] On the other hand, the PIA kinetics could be better tracked by averaging the signal between 530 to 600 nm (2.07 – 2.34 eV).

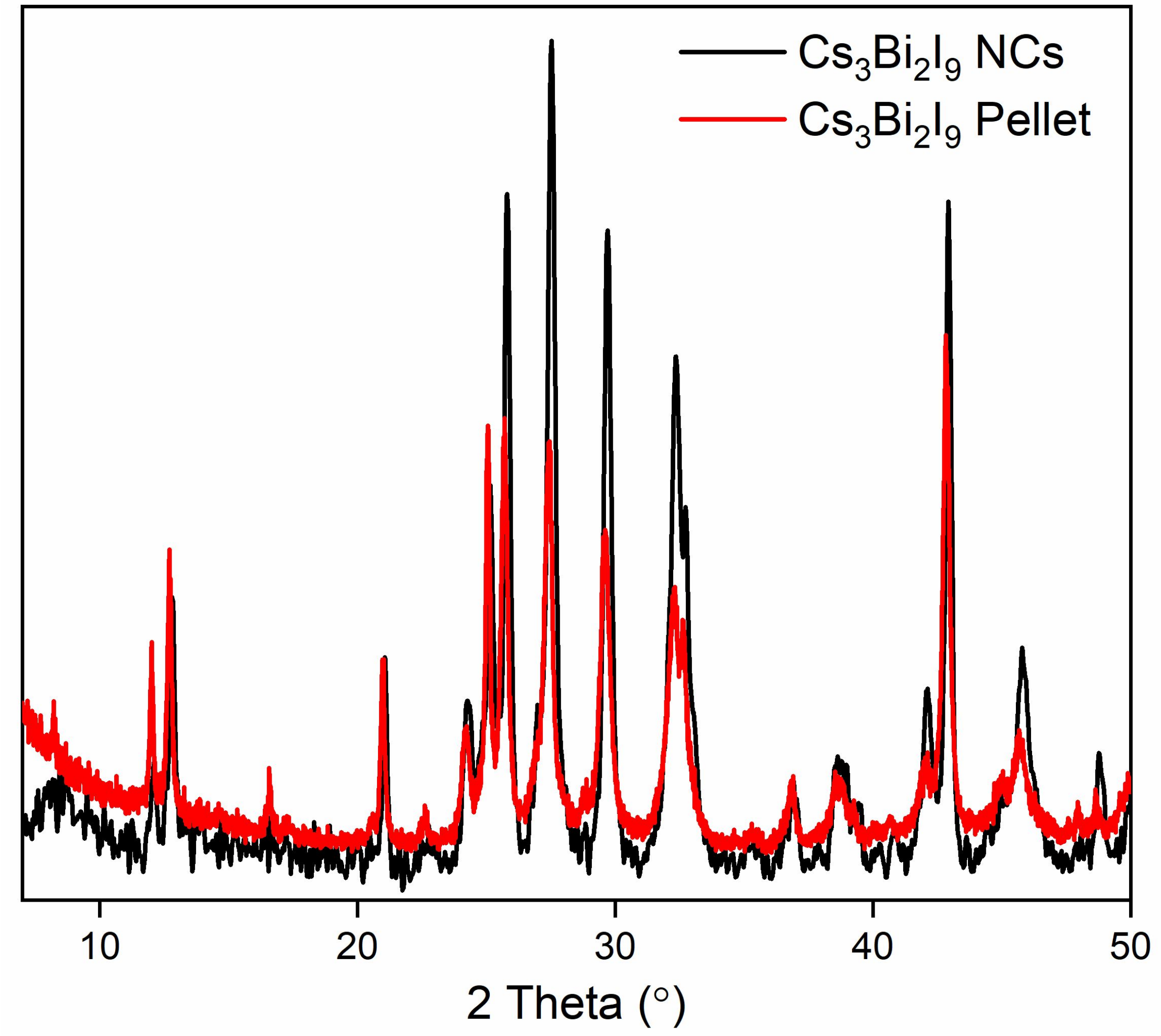

Figure S23. XRD patterns of the NCs (films) and pellets indicate that there are no significant changes before and after gentle grinding, confirming that the crystal structure is preserved.

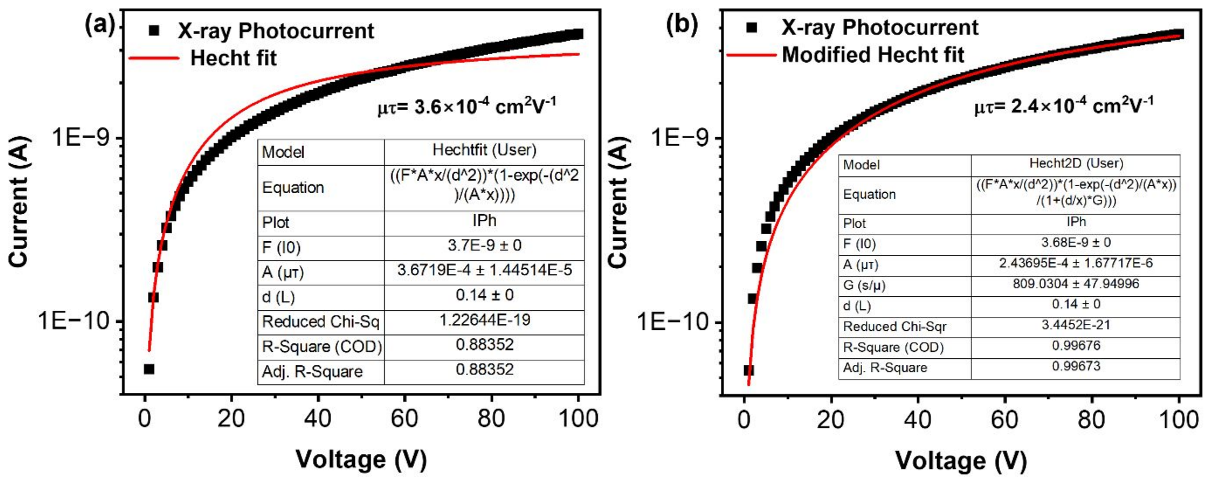

Figure S24. Fitting of the photocurrent using (a) conventional Hecht equation (b) modified Hecht equation.

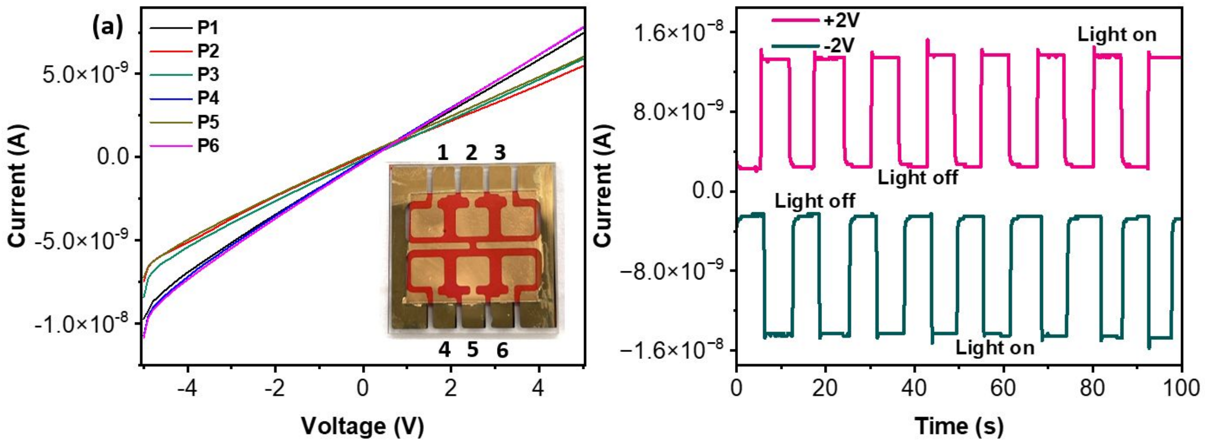

Figure S25. (a) Dark I-V of six FTO /Cs3Bi2I9 NCs thick film/Au pixels. (b) Temporal photocurrent response of the thick film device under illumination of 1sun light and applied bias of ±2 V.

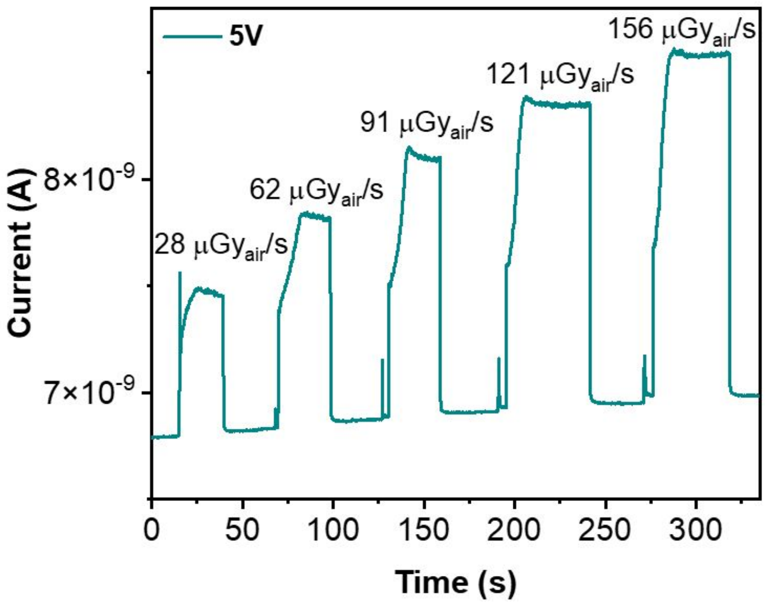

Figure S26. Temporal photocurrent response of the thick film detector under irradiation of X-ray with different dose rates and applied bias of 5V.

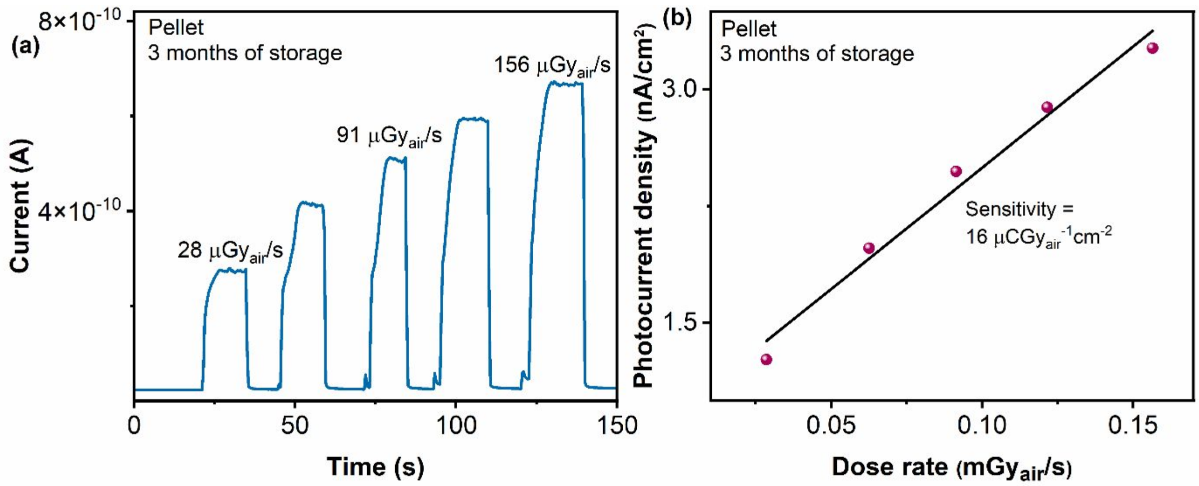

Figure S27. (a) Temporal photocurrent response of a $Cs_3Bi_2I_9$ NCs pellet under a 20 V applied bias at different dose rates after three months of storage. (b) Calculated sensitivity of the pellet detector.

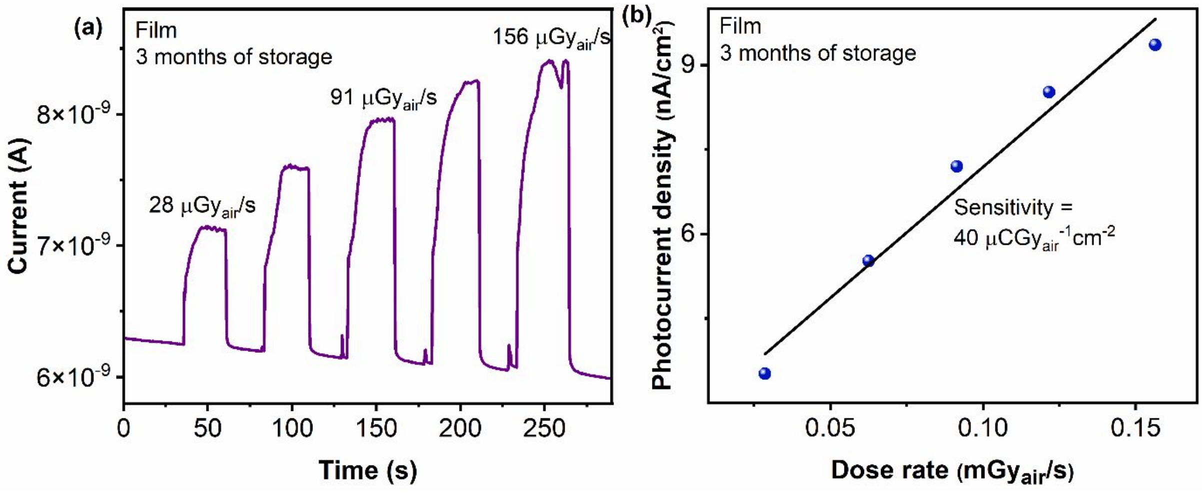

Figure S28. (a) Temporal photocurrent response of a $Cs_3Bi_2I_9$ NCs thick film under a 5 V (field of 1666 Vcm^{-1}) applied bias at different dose rates after three months of storage. (b) Calculated sensitivity of the thick film detector.

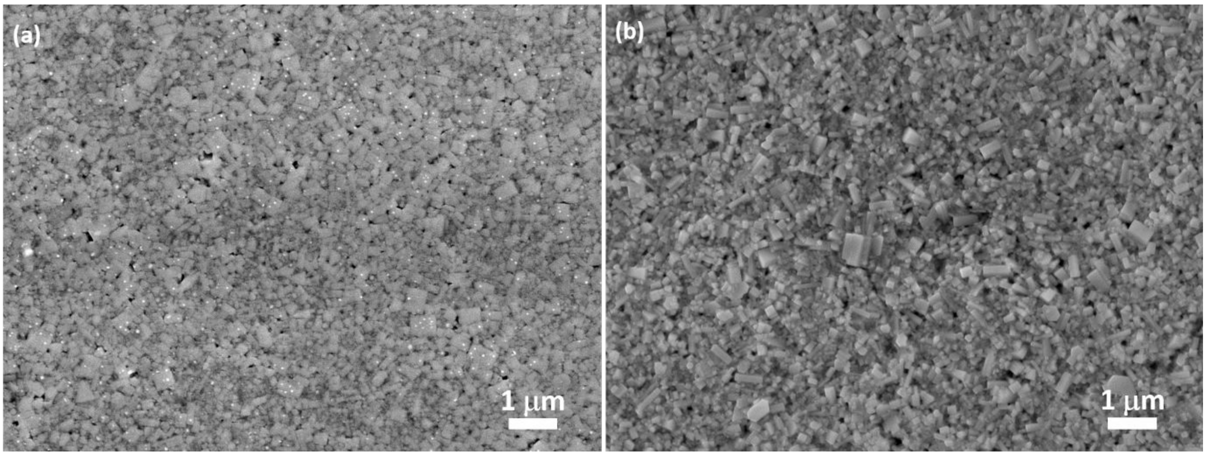

Figure S29. (a) SEM image of $Cs_3Bi_2I_9$ NCs pellet. (b) SEM image of $Cs_3Bi_2I_9$ NCs film.

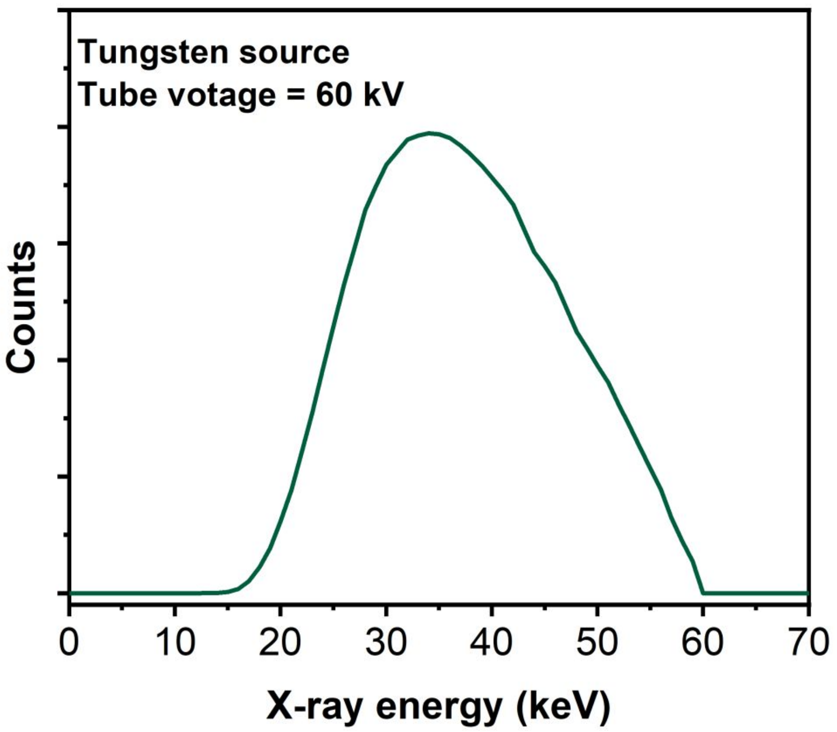

Figure S30. Simulated X-ray spectrum of a tungsten X-ray source operating at a tube voltage of 60 kV.

Table S1: Performance of the reported lead-free perovskite wafer and thick X-ray detectors.

Material	X-ray energy (acceleration voltage)	Active (material) area (cm^2)	μτ product ($cm^2 V^{-1}$)	Electric field (Vcm^{-1})	Sensitivity ($\mu CGy_{air}^{-1}cm^{-2}$)	Limit of Detection ($nGy_{air}^{-1} s^{-1}$)	Reference
a-Se polycrystalline film (1000 μm thick)	-	-	-	10^5	20	5500	[2]
$MA_3Bi_2I_9$ wafer	35.5 keVp	0.09	4.6×10^{-5}	2100	563	9.3	[3]
$Cs_2AgBiBr_6$ wafer with BiOBr	30 keV_p	(19.6)	5.5×10^{-3}	5000	250	95.3	[4]
$Cs_3Bi_2I_9$ wafer	(40 kV)	0.0314 (3.14)	1.67×10^{-4}	400	230.5	61.2	[5]
$Cs_2AgBiBr_6$ wafer	120 keVp	(8.64)	1.27×10^{-4}	1000	114	200	[6]
$Cs_3Bi_2I_9$ thick film	70 keVp	1.5 (6.25)	-	2400	127.2	7400	[7]
$MA_3Bi_2I_9$ thick film	30.6 keVp	-	3.89×10^{-5}	1360	35	140	[8]
$MA_3Bi_2I_9$ films	(40 kV)	-	1.6×10^{-6}	3000	100.2	98.4	[9]
$Cs_3Bi_2I_9$ NCs wafer	35 keV_p	0.2 (0.78)	2.4×10^{-4}	714	93	108	This work
$Cs_3Bi_2I_9$ NCs thick film	35 keV_p	0.25 (9)	-	1666	30.3	66	This work

REFERENCES

(1) Li, W.-G.; Wang, X.-D.; Liao, J.-F.; Jiang, Y.; Kuang, D.-B. Enhanced On–Off Ratio Photodetectors Based on Lead-Free Cs3Bi2I9 Single Crystal Thin Films. *Advanced Functional Materials* **2020**, *30*, 1909701.

(2) Kasap, S.; Frey, J. B.; Belev, G.; Tousignant, O.; Mani, H.; Laperriere, L.; Reznik, A.; Rowlands, J. A. Amorphous selenium and its alloys from early xeroradiography to high resolution X-ray image detectors and ultrasensitive imaging tubes. *physica status solidi (b)* **2009**, *246*, 1794-1805.

(3) Tie, S.; Zhao, W.; Xin, D.; Zhang, M.; Long, J.; Chen, Q.; Zheng, X.; Zhu, J.; Zhang, W.-H. Robust Fabrication of Hybrid Lead-Free Perovskite Pellets for Stable X-ray Detectors with Low Detection Limit. *Advanced Materials* **2020**, *32*, 2001981.

(4) Yang, B.; Pan, W.; Wu, H.; Niu, G.; Yuan, J.-H.; Xue, K.-H.; Yin, L.; Du, X.; Miao, X.-S.; Yang, X.; Xie, Q.; Tang, J. Heteroepitaxial passivation of Cs2AgBiBr6 wafers with suppressed ionic migration for X-ray imaging. *Nature Communications* **2019**, *10*, 1989.

(5) Bu, N.; Jia, S.; Xiao, Y.; Li, H.; Li, N.; Liu, X.; Yang, Z.; Zhao, K.; Liu, S. Inch-size Cs3Bi2I9 polycrystalline wafers with near-intrinsic properties for ultralow-detection-limit X-ray detection. *Journal of Materials Chemistry C* **2022**, *10*, 6665-6672.

(6) Li, W.; Liu, L.; Tan, M.; He, Y.; Guo, C.; Zhang, H.; Wei, H.; Yang, B. Low-Cost and Large-Area Hybrid X-Ray Detectors Combining Direct Perovskite Semiconductor and Indirect Scintillator. *Advanced Functional Materials* **2021**, *31*, 2107843.

(7) Chen, Y.-T.; Wen, Z.-X.; Lin, C.-F.; Li, M.-H.; Chen, P. Inorganic Cs3Bi2I9 lead-free halide perovskite film for large-area X-ray detector via low-cost ambient spray coating. *NPG Asia Materials* **2024**, *16*, 34.

(8) Xin, D.; Dong, S.; Zhang, M.; Tie, S.; Ren, J.; Lei, L.; Yu, P.; Zhu, J.; Zhao, Y.; Zheng, X. Nucleation Engineering in Sprayed MA3Bi2I9 Films for Direct-Conversion X-ray Detectors. *The Journal of Physical Chemistry Letters* **2022**, *13*, 371-377.

(9) Dong, S.; Xin, D.; Zhang, M.; Tie, S.; Cai, B.; Ma, Q.; Zheng, X. Green solvent blade-coated MA3Bi2I9 for direct-conversion X-ray detectors. *Journal of Materials Chemistry C* **2022**, *10*, 6236-6242.
